# How Tissue Mechanical Properties Affect Enteric Neural Crest Cell Migration

**DOI:** 10.1038/srep20927

**Published:** 2016-02-18

**Authors:** N.R. Chevalier, E. Gazguez, L. Bidault, T. Guilbert, C. Vias, E. Vian, Y. Watanabe, L. Muller, S. Germain, N. Bondurand, S. Dufour, V. Fleury

**Affiliations:** 1 Laboratoire Matière et Systèmes Complexes, Université Paris-Diderot/CNRS UMR 7057, 10 rue Alice Domon et Léonie Duquet, 75013 Paris, France; 2 UMR144, CNRS-Institut Curie, 26, rue d’Ulm, 75248 Paris cedex 05, France; 3 Collège de France, Center for Interdisciplinary Research in Biology (CIRB), Paris, F-75005, France; 4 INSERM, U1050, Paris, F-75005, France; 5 CNRS, UMR 7241, Paris, F-75005, France; 6 INSERM, U1016, Institut Cochin, Paris, France; 7 CNRS, UMR8104, Paris, France; 8 Université Paris Descartes, Sorbonne Paris Cité, Paris, France; 9 INSERM U955, Equipe 11, F-94000 Créteil, France

## Abstract

Neural crest cells (NCCs) are a population of multipotent cells that migrate extensively during vertebrate development. Alterations to neural crest ontogenesis cause several diseases, including cancers and congenital defects, such as Hirschprung disease, which results from incomplete colonization of the colon by enteric NCCs (ENCCs). We investigated the influence of the stiffness and structure of the environment on ENCC migration *in vitro* and during colonization of the gastrointestinal tract in chicken and mouse embryos. We showed using tensile stretching and atomic force microscopy (AFM) that the mesenchyme of the gut was initially soft but gradually stiffened during the period of ENCC colonization. Second-harmonic generation (SHG) microscopy revealed that this stiffening was associated with a gradual organization and enrichment of collagen fibers in the developing gut. *Ex-vivo* 2D cell migration assays showed that ENCCs migrated on substrates with very low levels of stiffness. In 3D collagen gels, the speed of the ENCC migratory front decreased with increasing gel stiffness, whereas no correlation was found between porosity and ENCC migration behavior. Metalloprotease inhibition experiments showed that ENCCs actively degraded collagen in order to progress. These results shed light on the role of the mechanical properties of tissues in ENCC migration during development.

Neural crest cells (NCCs) are essential to vertebrate development. This highly migratory and multipotent population of cells gives rise to craniofacial structures, cardiac and neuroendocrine derivatives, melanocytes, glial cells and neurons of the peripheral nervous system, as well as the intrinsic innervation of the digestive tract, the enteric nervous system (ENS). Different methods have been used to visualize NCCs and their derivatives, ranging from Le Douarin’s historical chick-quail neural tube graft method^1^ to the more recent targeting of NCCs with reporter fluorescent proteins, making the dynamic follow-up of their migration within the embryo possible^2^,^3^. Depending on their rostro-caudal level along the neural tube, the cranial, vagal, trunk and sacral NCCs follow different paths. Enteric NCCs (ENCCs) mostly consist of vagal NCCs that enter the foregut and migrate through the gut mesenchyme toward the distal hindgut. Sacral NCCs entering the distal hindgut and colonizing the gut tissue caudo-rostrally make a small contribution to the ENS^4^,^5^. ENCC colonization occurs at a stage during which the gut tissue increases in length and differentiates^6^. ENCCs always migrate in a 3D environment composed of other cell types and the extracellular matrix (ECM). Other NCC populations have been found to crawl on planar surfaces, such as the collagen-rich basal lamina. Such behavior is observed, for example, during the dorso-ventral migration of trunk NCCs along the neural tube^5^.

Several signaling pathways involved in correct ENS development have been elucidated in recent decades. GDNF (glial-derived neurotrophic factor) is a protein secreted by the mesenchymal cells of the gut^7^ that binds to the RET membrane receptor and co-receptor GFRalpha on ENCCs and acts as a chemoattractant^8^ and a differentiation signal. The endothelin-3 (edn3)/EDNRB ligand-receptor pair plays an essential role in maintaining the ENCC population in a proliferative and undifferentiated state. More recently, adhesion molecules (β1-integrin, N-cadherin, L1-CAM) have been shown to contribute to ENS development by regulating ENCC migratory behavior^9^,^10^,^11^,^12^,^13^. Our understanding of the molecular mechanisms controlling NCC migration has thus deepened, and we now have a more detailed molecular description of the main ECM components through which NCCs migrate^11^,^14^,^15^.

In addition to the role of local biochemical factors, the mechanical properties and geometric nature of the cellular environment have been shown to dictate morphogenesis by changing cell fate, shape and migratory behavior, and these parameters act in synergy with growth factors, soluble peptides and the ECM^16^,^17^,^18^,^19^,^20^,^21^. Environmental stiffness has been shown to have a large effect on adhesion, proliferation, migration and differentiation in various cell lines^22^,^23^,^24^, but the ways in which ENCCs regulate their adhesion and migration in response to this parameter have not been investigated and the mechanical properties (elasticity) of the developing gut during ENCC colonization are unknown.

We measured, *ex vivo*, the bulk and micron-scale mechanical properties of chick and mouse embryonic gut at different locations along the rostro-caudal axis (pre-umbilical and post-umbilical midgut, caecum and hindgut) spanning developmental times E4–E9 for chicks (the complete gut colonization period) and E12.5 to E14.5 for mice (the hindgut colonization period). We found that ENCC migration occurred in soft gut tissues that progressively stiffened during colonization. By second-harmonic generation (SHG) imaging of gut sections, we determined the density and distribution of fibrillar collagen along the rostro-caudal axis of the developing gut and showed that the collagen fibers of the ECM accounted for almost half the elastic modulus of the gut tissue. We demonstrated, in *ex-vivo* experiments performed in 2D and 3D environments, that ENCC migration was modulated by the mechanical properties of the environment. Our findings thus demonstrate that the regulation of ENCC migration is dependent on ECM stiffness and structure, and shed light on the role of the mechanical properties of gut tissue during ENS ontogenesis.

## Results

### Embryonic gut mesenchyme stiffness during colonization by enteric neural crest cells

**Figure 1:**
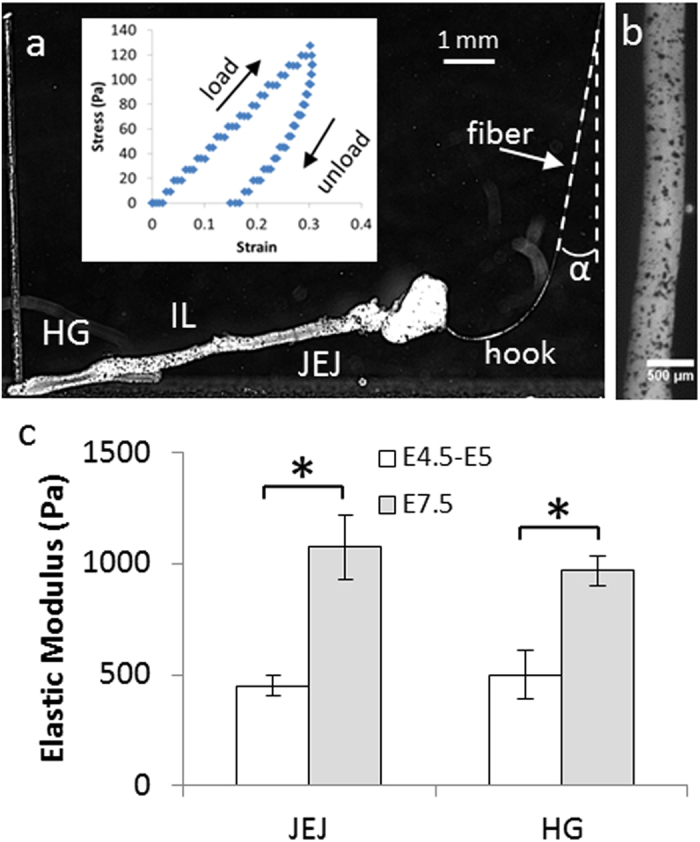
Bulk tensile stiffness of embryonic chick gut. (**a**) Side view of a tensed E5.5 gut. The end of the fiber (total length ~10 cm) is seen on the right (white arrow), α: fiber deflection angle, HG: hindgut, IL: ileum, JEJ: jejunum. Inset: Typical stress-strain load and unload curve for E5.5 chick gut ileum. The slope of the loading curve yields the modulus. Creep is observed, as the gut does not return to its initial length immediately after unloading. (**b**) High-magnification view of a gut sprinkled with carbon particles, which serve as deformation tracers for particle image velocimetry (PIV), (**c**) Change in the elastic modulus (Pa) in the jejunum and hindgut regions of the chick gut at the jejunum colonization stage (E4.5–E5, *n* = 5) and during hindgut colonization (E7.5, *n* = 5). Error bar length is the standard deviation across samples. A star indicates a significant difference (p < 0.05, two-tailed Mann-Whitney test).

We devised a simple uniaxial tensile test based on glass fiber deflection^25^,^26^ to determine the bulk elastic modulus of chick embryonic gut (Fig. 1a,b, detailed description in Materials & Methods). We simultaneously measured the angular deflection of the fiber, to assess the tensile force applied to the digestive tract, and the resulting deformation in the jejunum (proximal midgut), ileum (distal midgut), and hindgut. The slope of the line for the stress-strain data in each region corresponds to the local tensile elastic modulus (Fig. 1a inset). All regions were deformed simultaneously by the same force ramp, providing a reliable way of comparing elastic modulus variations along the rostro-caudal axis of the gut.

Stress-strain characteristics were linear up to at least ~20% deformation (Fig. 1a inset). The elastic modulus was not significantly dependent on strain rate, so long as strain rate remained well above the viscoelastic relaxation rate of the gut. The embryonic gut stiffened (increased elastic modulus) during its development from E4.5 to E7.5 (Fig. 1c). The elastic modulus values we found (200–1000 Pa) are consistent with the results obtained for other embryonic tissues in other studies with micropipette aspiration techniques^27^. These previous studies also reported developmental stiffening of the heart. The elasticity of E8 embryonic chick midgut (jejunum) was measured by Savin *et al.*^28^ using a magnet and grafted steel bead system, they find a modulus value of 4000 Pa whereas our measurements indicate 1074 ± 144 Pa in the jejunum at E7.5. This discrepancy might be due to differences in the buffers used for the tensile test (phosphate-buffered saline here, Krebs-Ringer in the work of Savin). As Krebs-Ringer contains glucose and magnesium, this might increase the active contribution of muscles to the tensile stiffness (muscles are already well developed in the jejunum at E8). Differences relating to chick species (breed of JA57 cocks and I66 hens here, White Leghorn in the work of Savin *et al.*) also cannot be excluded. We used the detailed chronology of ENCC migration in the chick established by Goldstein & Nagy^29^ to infer the developmental stage at which the ENCC migratory front crossed the jejunum, ileum and hindgut. At the stages at which ENCCs are colonizing the midgut (E4.5–E5), they encounter a ~450 Pa environment, and when they migrate through the hindgut (E7.5), they encounter a tissue with a higher tensile elastic modulus of ~1000 Pa.

**Figure 2:**
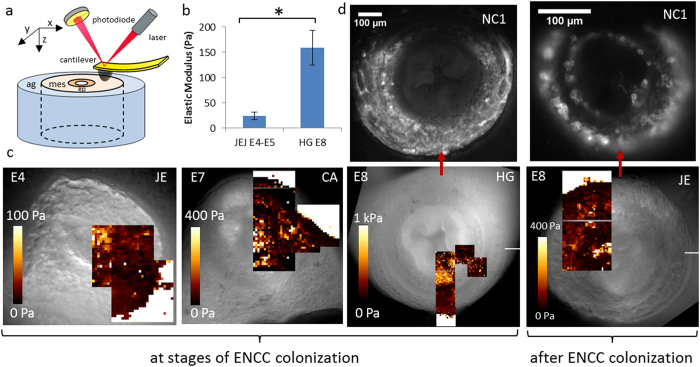
AFM elasticity maps of freshly prepared sections of gut embedded in agarose gels. (**a**) Scheme of the experimental setup. The AFM cantilever (image courtesy of W.Roos) indents a transverse section of gut (mes: mesenchyme, ep: epithelium) embedded in an agarose (ag) gel along the Z direction. After each indentation, the cantilever is displaced in the XY plane to generate stiffness maps. (**b**) Mean mesenchyme stiffness deduced from AFM maps at the jejunum (E4.5–E5, *n* = 4 different guts) and hindgut (E8, *n* = 4 different guts) colonization stages. For each gut section, the mesenchyme stiffness was calculated by averaging over 2–5 100 × 100 μm^2^ maps, and each map comprises ~50–400 pixels (each pixel yields one local modulus value). Error bar length is the standard deviation across samples. A star indicates a significant difference (p < 0.05, two-tailed Mann-Whitney test). (**c**) E4-JE, E7-CA (caecum) and E8-HG are sections at stages during ENCC colonization. E8-JE corresponds to a region that has already been colonized by ENCCs. Each AFM map is a 100 × 100 μm square, typically with a 5 μm step (i.e., 20 × 20 px). Up to six maps were obtained per sample.The white region on AFM maps located around the gut sections corresponds to the stiff agarose gel in which the sample is embedded. (**d**) Post-AFM NC1 immunostaining of E8-HG and E8-JE sections showed the ENCCs to be located at the outer periphery of the gut. The dark region at the top of these images results from incorrect placement of the microscope diaphragm.

One limitation of the uniaxial tensile test is that it yields the mean bulk stiffness, but does not provide information about local elasticity or its variation within the gut mesenchyme, where ENCCs migrate. We addressed this point by atomic force microscopy (AFM) indentation, in force spectroscopy mode, to probe fresh transverse gut sections (Fig. 2a) at different developmental stages. Each indentation probed the elasticity of about 3–10 cells. We superimposed elasticity maps onto the corresponding microscopy images of the gut sections analyzed at stages E4–E8 (Fig. 2c), both in regions recently invaded by ENCCs and in regions in which the ENCCs had already established themselves to form the myenteric and submucosal plexi. Figure 2d presents immunostaining for NC1 (a marker of ENCCs) of the E8 hindgut and E8 jejunum sections mapped by AFM. We observed no systematic large-scale gradients or structures of differing stiffness within the mesenchyme of the gut for ages E4–E7. In the jejunum at E8 (Fig. 2c), the muscularis appeared to be stiffer than the mucosa. The more central regions of the gut sections were stiffer (by a factor of 2–8) than the more peripheral regions. These stiffer regions were often correlated with the location of the epithelium. The mean AFM stiffness of the mesenchyme increased with age (Fig. 2b,c); the mesenchyme stiffness is higher by the time the ENCCs colonize the hindgut than it was when they were colonizing the midgut (Fig. 2b). These results corroborate, at a smaller scale, those found by bulk tensile testing (Fig. 1c).

The elastic modulus values deduced from AFM indentation (20–200 Pa) were consistent with AFM measurements performed by Henkels *et al*. on chicken embryo blastula explants at the gastrulation stage. However, they were an order of magnitude lower than the values deduced from uniaxial tensile testing (200–1000 Pa). We believe that this difference may reflect differences between the tests: the tensile test measures the section-averaged tensile modulus, whereas AFM measures the local compressive modulus. Longitudinal fibrous structures (e.g. collagen, elastin), which are in a tensile state in the traction assay, contribute less to the elasticity of the organ when under compression (AFM). The cutting of transverse sections probably also disrupts the local mechanical properties of the gut, by cutting fibers/cell connections and damaging cells at the surface. Possible damage due to cutting does not however affect the conclusions we come to concerning the elasticity distribution within a section and its time evolution for developing organs. The maps presented in Fig. 2 represent, to the best our knowledge, the first force spectroscopy images of live embryonic tissue sections combined with immunohistochemical mapping, and the first comparison of AFM elasticity measurements with another independent method (tensile testing) for this type of tissue.

### Developmental stiffening of the gut is linked to gradual enrichment in collagen fibrils and the organization of these fibrils

Collagen plays a crucial role in ensuring tissue mechanical integrity, particularly in the gut wall^30^. We assessed the contribution of collagen to the elastic modulus, by measuring the tensile elastic modulus of E5.5 guts (*n* = 3) before 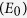 and after 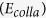 treatment with collagenase for 10 minutes. The estimated contribution of collagen to the elastic modulus 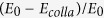 was 43.3 ± 7.7%. This value is consistent with findings on the effect of collagenase on embryonic heart tissues^27^ and confirms that collagen is one of the important contributors to tensile stiffness. Developmental stiffening may, therefore, be linked to a change in collagen production and distribution within the developing gut. We used second-harmonic generation (SHG) microscopy to assess the endogenous signal from collagen I and III fibers, the main sources, together with myosin, of SHG in biological tissues^31^,^32^. The SHG technique has been reviewed in recent work^33^,^34^,^35^ and is often used to assess the role of fibrillar collagen on biomechanical properties of biological tissues^36^,^37^,^38^. For dermal samples, SHG signal has been shown to co-localize with the fluorescence of collagen I immunhistochemistry^39^. One advantage of SHG microscopy is that it does not require immunolabeling or dehydration of samples; fibrous collagen SHG signal intensity can therefore be quantitatively obtained without any artefacts arising from differences in antibody binding/penetration/application time across samples. Contrary to electron microscopy, SHG also does not require dehydration of the tissue, which would clearly affect the native collagen organization. Collagen I mapping along NCC pathways has, to our knowledge, previously been performed only by immunohistochemical methods, at earlier stages of chick embryo development^14^.

**Figure 3:**
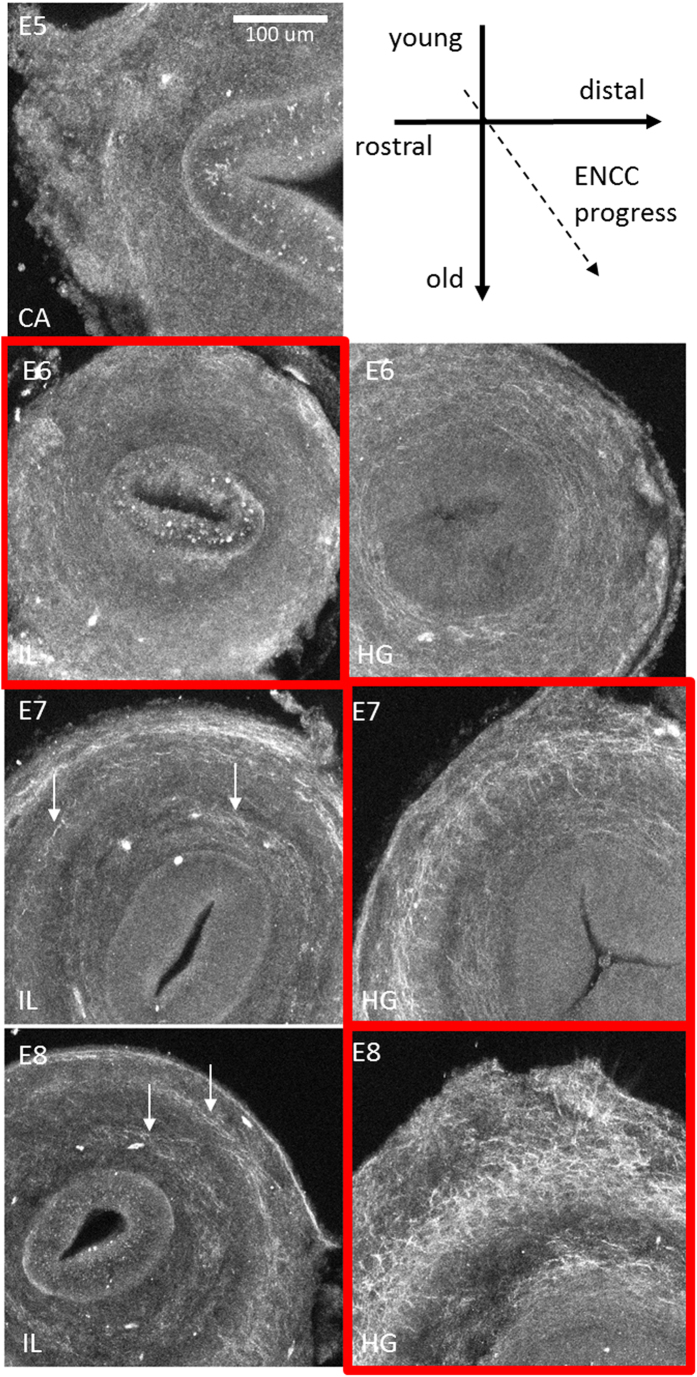
Second-harmonic generation (SHG) images of chick gut sections at different developmental stages (E5–E8) and for different regions along the rostro-caudal axis. IL = ileum. CA = caecum, HG = hindgut. The images are maximum *z-*projections of 3 μm-step *z*-stacks over a total depth of 100 μm from the section surface. Red boxes surround sections corresponding to the approximate position of the ENCC front at the developmental time point concerned. All the acquisition parameters of the SHG microscope were kept constant between samples so that SHG signal intensities could be compared across ages and gut regions. White arrows in IL E7–E8 indicate rings of collagen fibers not present at earlier stages. The scale bar is provided at the top right and is the same for all images. Another sample comparing SHG at E6 and E8 in the hindgut is presented in Figure S12.

We obtained spatiotemporal SHG pictures of embryonic chick gut sections (Fig. 3). The boxes outlined in red indicate sections located at the approximate position of the ENCC migratory front at these time points in development. During the colonization of the ileum and caecum by ENCCs (E5–E6), the SHG signal intensity was uniform in the mesenchyme (consistent with the results of a previous study^14^ on E4 chick gizzard). This uniform signal may correspond either to non-specific background signal (noise) or to short, uniformly distributed collagen fibrils. When the ENCCs reached the hindgut at about E7, the collagen fibers in this region were found to form two rings (Fig. 3, E7–8, HG), an inner ring located next to the epithelium (in the region of the submucosa/lamina propria) and a more peripheral ring, presumably corresponding to the developing circular muscle layer (muscle tissue is already present when the ENCCs reach the chick hindgut). The collagen fiber density increased in the HG from E6 to E8. In the colonized zone of the gut (E7–8, ileum), distinct, circularly arranged collagen fibrils concentrated to form two rings (Fig. 3, white arrows), that were not seen at earlier stages in this region (E5–6, ileum).

### Developmental stiffening in mice

**Figure 4:**
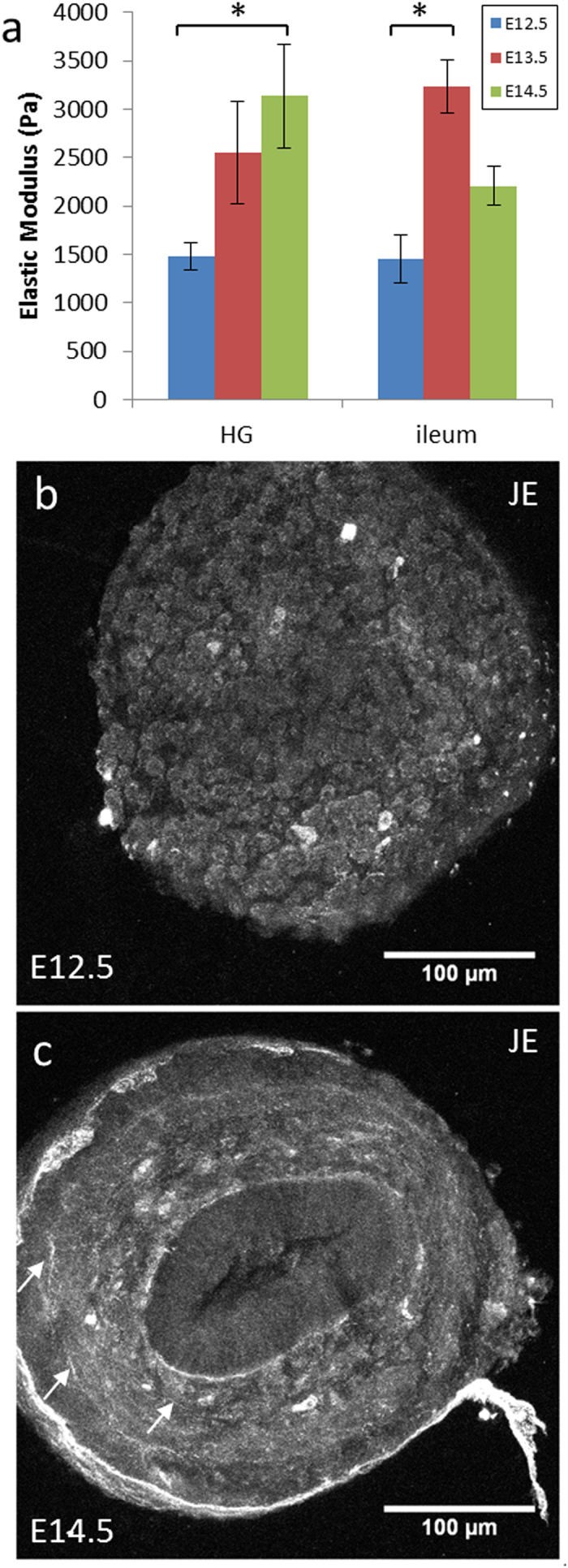
Stiffness and collagen in mouse guts. (**a**) Changes in the genotype-averaged elastic modulus of mouse guts from E12.5 (*n* = 3 Edn3^ls/ls^, *n* = 3 Edn3^ls/+^, *n* = 2 wild-type), E13.5 (*n* = 3 Edn3^ls/+^, *n* = 2 wild-type) and E14.5 (*n* = 2 Edn3^ls/+^, *n* = 1 wild-type), for the hindgut and ileum. The error bars indicate the SEM. A star indicates a significant difference (p < 0.05, two-tailed Mann-Whitney test). (**b**) Representative SHG *z*-stack maximum projections for E12.5 and E14.5 jejunum (JE), in the same acquisition conditions as in Fig. 3. White arrows point at circularly arranged collagen fibers at E14.5, that are not present at E12.5. The complete SHG data set is shown in the Figure S13.

Abnormally high levels of collagen IV and laminin have been reported in the guts of lethal spotted (Edn3^ls/ls^) mice^40^. These mice display colonic aganglionosis, one of the phenotypic outcomes of Hirschsprung disease. As collagen IV and laminin could potentially contribute to the stiffening of the embryonic gut, we compared the guts of Edn3^ls/ls^ mice with heterozygous (Edn3^ls/+^) and wild-type (Edn3^+/+^) mice at E12.5, in tensile tests. The average moduli of Edn3^ls/ls^ in the hindgut and ileum were within the error bar of the average moduli measured for Edn3^ls/+^ or Edn3^+/+^ mice at stage E12.5 (Figure S1). When we pooled the different genotypes, we found that mouse gut tissue stiffened (Fig. 4a) over the course of development, from E12.5 (ENCC migratory front at the proximal hindgut) to E14.5 (gut fully colonized). Hindgut stiffness significantly increased from 1.5 kPa at E12.5 to 3.2 kPa at E14.5. SHG images showed distinct, circularly arranged collagen fibers at E14.5 (white arrows in Fig. 4c) whereas no distinct fibers could be seen at E12.5 (uniform signal, Fig. 4b). These results on mice are similar to those presented for chick guts (Figs 1 and 3). They also reveal that changes in collagen IV and laminin in these mutant mice do not have an impact on tissue stiffness (within the resolution of our measurement, ± 465 Pa).

Having characterized the micromechanical properties of the embryonic mesenchyme during gut colonization by ENCCs, we investigated the effect of changes in the stiffness of the environment on ENCC migration in two and three dimensions.

### ENCCs can adhere to and migrate on soft 2D substrates

**Figure 5:**
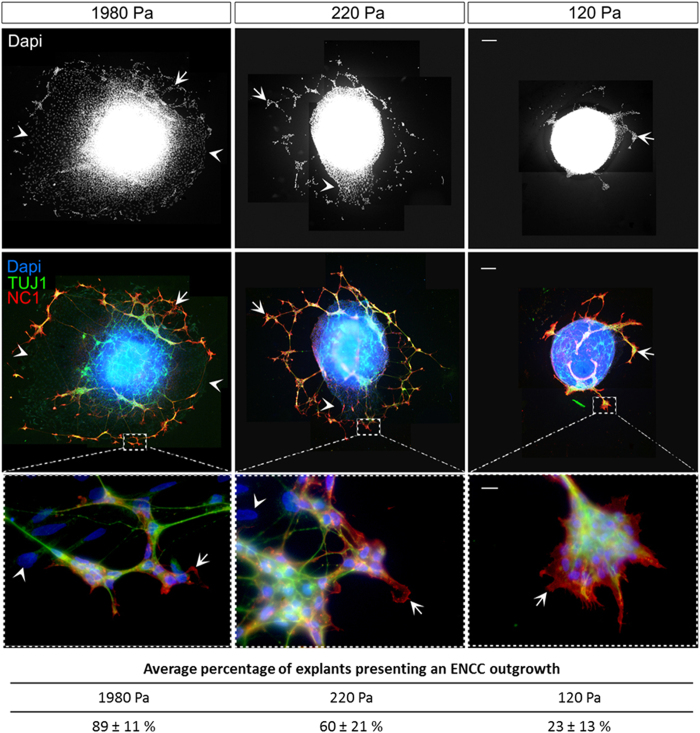
2D migration assay of ENCCs on PAA gel substrates of various stiffnesses. The PAA gels were coated with covalently attached FN at a fixed density, to facilitate the adhesion of ENCCs. Gut explants were cultured overnight, fixed and immunostained for the NCC marker (NC1, red) and a neuronal marker (TUJ1, green), and labeled with DAPI (nucleus, blue). The upper panels show Dapi staining of the cell outgrowth around the explant the arrows and arrowheads. The middle panels show the merge images of Dapi, NC1 and TUJ1 stainings revealing the ENCC (NC1+/TUJ1+/Dapi+, arrows) and mesenchymal cells (NC1−/TUJ1−/Dapi+, arrowheads), Bar = 100 μm. The lower insets show ENCCs (arrows) and mesenchymal cell morphology (arrowheads) at a higher magnification, Bar = 10 μm. The lower panel shows the mean percentage ( ± SEM) of explants displaying ENCC outgrowth, averaged over the three independent experiments performed. The total number of explants analyzed was 10 for 1920 Pa, 19 for 220 Pa and 21 for 120 Pa.

It has been clearly shown that cell adhesion and migratory properties in two dimensions can be modulated by substrate stiffness^24^. However, it remains unclear how ENCCs perceive and respond to the rigidity of their environment. We cultured chick embryonic midgut explants (at stage E5.5/E6.5) overnight on glass and on 2D PDMS substrates of different stiffnesses (28 kPa, 15 kPa and 1.5 kPa) coated with fibronectin (FN). We compared the capacity of cells to escape from the explant and migrate on these substrates. The cell outgrowths, composed of ENCC (NC1+/TUJ1+/Dapi+) and mesenchymal cells (NC1−/TUJ1−/Dapi+), were similar for all stiffnesses considered (Figure S2), indicating that ENCCs and mesenchymal cells were able to adhere and to migrate equally well in the range 1.5 kPa to GPa (glass). We then cultured gut explants on FN-coated polyacrylamide (PAA) gels^41^ of gut mesenchyme-like stiffness (see Fig. 1): 1980 Pa, 220 Pa and 120 Pa (Fig. 5). Both ENCCs (arrows) and mesenchymal cells (arrowheads) are able to migrate from the gut explant when cultured on the 1980 Pa PAA gel (Fig. 5, top and middle left panels). On 220 Pa gels, the cell outgrowth was less extended and mostly composed of ENCC with very few mesenchymal cells (Fig. 5, top and middle central panels, arrows and arrowheads respectively). On the softest gel (120 Pa) in contrast, the great majority of explants displayed no cell outgrowths (Fig. 5, table) or very reduced ones with only ENCCs (Fig. 5, top and middle right panels, arrows).

ENCC morphology was similar on 1980 Pa and 220 Pa substrates. The ENCCs displayed extended lamellipodia, revealing their capacity to adhere and spread efficiently on these substrates (Fig. 5, high magnification in the lower left and central panels, arrows). On 120 Pa PAA gels, ENCCs were more clustered but some of them exhibited protrusions and lamellipodia at the periphery of clusters indicating that ENCCs are able to adhere and spread (Fig. 5, lower right panel, arrow). Thus, ENCCs can interact with and migrate on substrates of stiffnesses similar to those measured for the embryonic gut at stage of ENCC colonization (220 Pa). The mesenchymal cells however could barely escape from the gut explants cultured on substrate rigidities below 1500 Pa, indicating an alteration of both their adhesion and migration capacities under this threshold.

### ENCC migratory front speed in 3D gels decreases with increasing matrix stiffness

**Figure 6:**
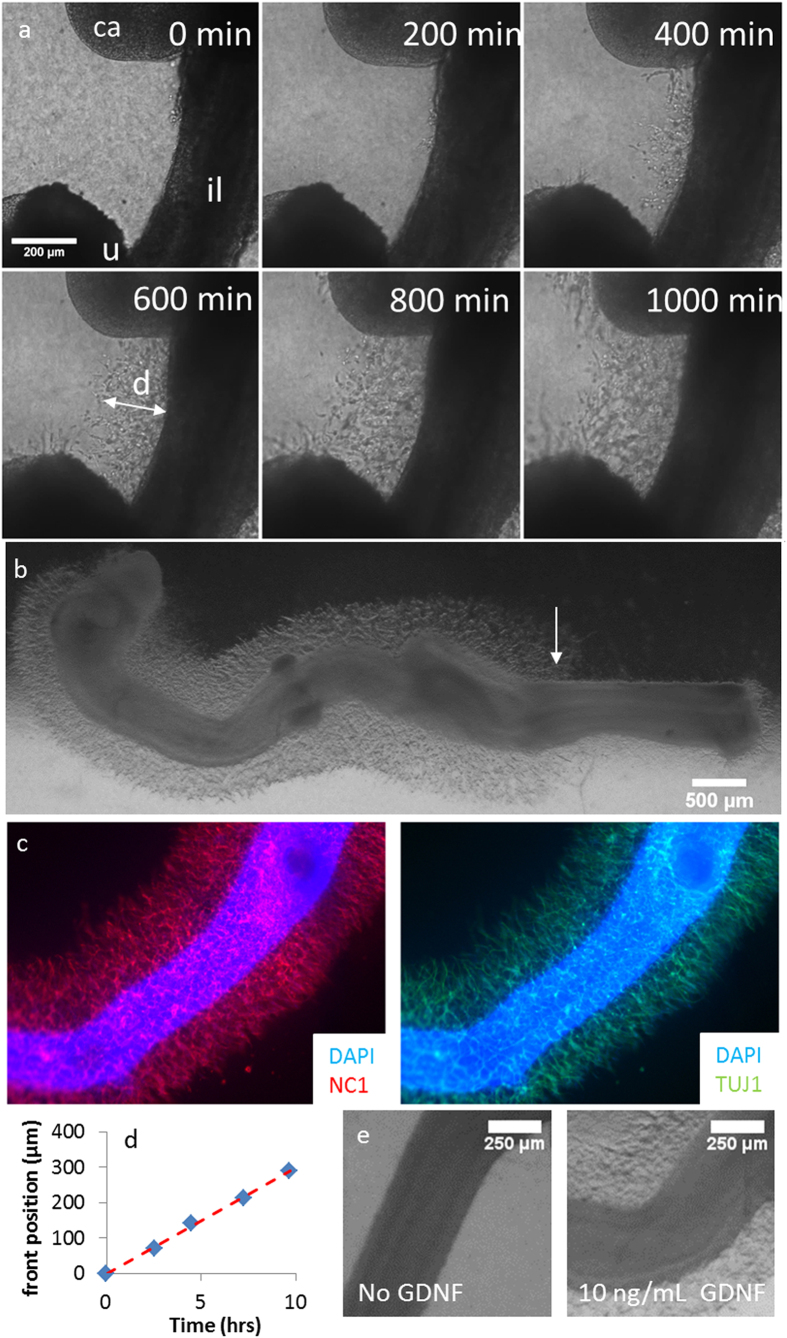
ENCCs from chick gut invading a 3D collagen matrix containing GDNF. (**a**) Extracts from time-lapse Movie 1 (see Online Material), E6 chick gut embedded in a 660 Pa gel supplied with DMEM and 10 ng/mL GDNF, on the stage of an inverted microscope maintained at 37 °C. Time is counted from the moment the gut (E6) was imaged (i.e., approximately ~1 h after embedding in the gel). il: ileum, ca: caecum, u: umbilicus. (**b**) Wider field view of the ENCC halo surrounding a gut in the same conditions, after 48 h of culture, left: rostral, right: distal. The distance traveled by ENCCs is greatest at the position of the ENCC migratory front in the gut, i.e., at the entrance of the hindgut at this stage (arrow). This distance is shorter at more rostral positions, yielding the overall pear shaped pattern observed in all cultures. (**c**) DAPI, NC1 and TUJ1 fluorescence staining of a gut in a 1500 Pa gel (gel no. 4) after 72 h of culture. All cell outgrowths labeled (*n* = 7) showed similar patterns of staining for NC1 and TUJ1. (**d**) Mean distance *d* of the ENCC migration front from the gut wall as a function of time extracted from (**a**). (**e**) Comparison of ENCC migration from E5.5 midguts placed in a 660 Pa gel after 24 h of incubation without GDNF (left) or with 10 ng/mL GDNF (right). See Figure S5 for the full concentration trial.

As ENCCs migrate in a 3D environment, we carried out a 3D migration test by embedding the duodenum to distal colon segment of embryonic chick guts (E5.5–6) in collagen gels (see Materials & Methods) supplied with the ENCC chemoattractant GDNF and nutrients. Figure 6a (excerpts from time-lapse Movie 1, available online) shows cells emerging from the gut wall and penetrating the collagen gel within hours after the start of incubation. Figure S3 shows that ENCCs are present both below and above the plane of the gut, indicating that they have invaded the gel surrounding the gut in all directions. Figure 6b shows a lower magnification view of the same gut after 24 h of culture. The cells that have migrated from the midgut form a halo. Immunolabeling of the samples for TUJ1 and NC1 (Fig. 6c) confirmed that these cells were ENCCs. The breadth of this halo increased rostro-caudally (Fig. 6b), reaching a maximum close to the position of the ENCC migratory front within the gut (white arrow Fig. 6b). In older guts (E9.5), the ENCCs form a cone (Figure S4d, arrow) extending in the rostro-caudal direction, confirming that the more rostral cells depart later, possibly because these more mature cells are less sensitive to GDNF signaling. Low-magnification time-lapse imaging (Movie 2) revealed that the shape of the halo resulted from the more distal cells leaving the gut earlier than the more rostral cells. No cells leaving the hindgut wall were observed, even in guts old enough for this part of the digestive tract to be already colonized. However, cells were observed leaving the cloaca (Movie 2). During the first 10 h of the experiment, ENCCs were found to advance at a mean speed of ~30 μm/h (Fig. 6d) in a 660 Pa gel. The stiffness of the 660 Pa gel is similar to that of the chick embryonic mesenchyme (Fig. 1), and the speed of migration measured in this gel was similar to that *in vivo* in chick^42^ or mouse guts^3^. Although differences in the mechanisms regulating ENCC migration in collagen gels compared to *in vivo* and intact gut segments grown *ex vivo* have been reported^7^,^43^,^44^,^45^, collagen gels do offer an opportunity to examine ENCC migration in a 3D environment. PIV tracking of the displacements of small particles embedded in the gel surrounding the gut showed that ENCCs pulled on the collagen gel to migrate (Figure S5). No migration occurred in the absence of GDNF (Fig. 6e), consistent with the known chemotactic effect of this molecule on ENCC^8^, but it should be stressed that NCCs at an earlier stage migrate from the neural tube in collagen gels without the addition of chemoattractant^46^,^47^. The density of the ENCC migratory front and its mean distance from the gut wall were found to increase as GDNF concentration increased from 0 to 10 ng/mL; further increases in GDNF concentration did not stimulate ENCC migration further (Figure S6).

**Figure 7:**
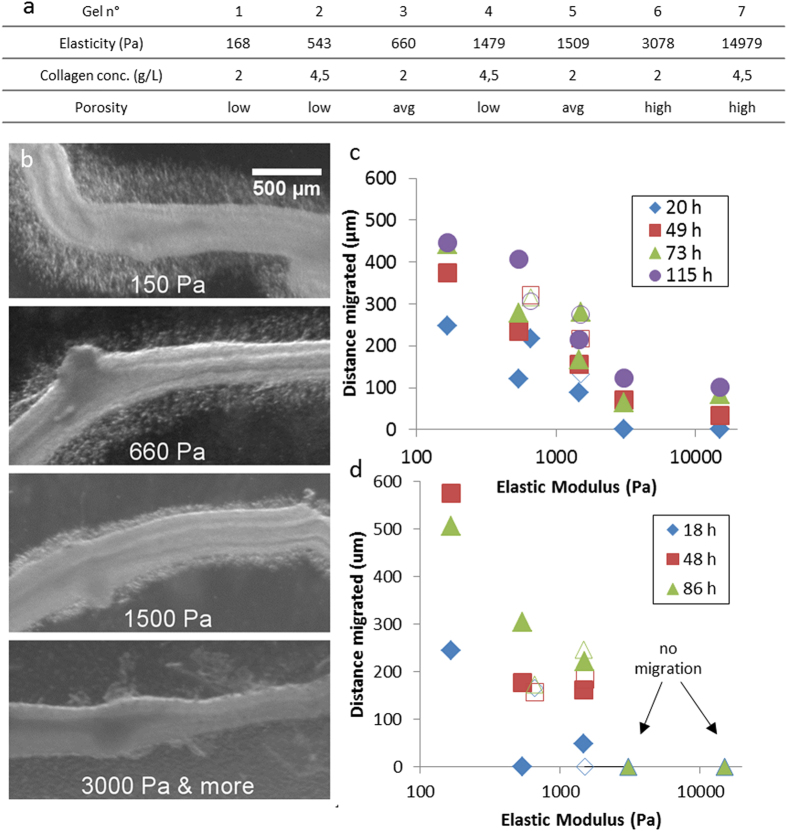
Effect of gel stiffness on ENCC migration. (**a**) Characteristics of the seven collagen gel formulations considered in this work, sorted in order of increasing Young modulus. (**b**) Migration halo around E6 chick guts embedded in gels of increasing stiffness, after 18 h of incubation. (**c**,**d**) Mean distance of the migration front from the gut wall as a function of gel elastic modulus at different times for two independent experiments. Error bars are smaller than the symbol width. For the two pairs of gels (2&3 and 4&5) with very similar stiffnesses but different porosities, the more porous gel is indicated by an open symbol. No migration was seen over an 86 h period for gels 6&7 in (**d**).

Porosity and gel stiffness were controlled by modifying collagen concentration, gelling temperature and the final pH of the gel (Figure S7). Seven gels spanning physiological bulk mesenchyme stiffness values (Figs 1, 4) from 150 Pa to 15 kPa were thus obtained. We imaged the structure of the gels by SHG microscopy (Figure S6) and assigned the gels to three porosity groups (low, average, high) on the basis of the fraction of the area occupied by pores greater than 3 μm in diameter: fewer than 25% (low porosity) between 25 and 40% (average) and more than 40% (high). The characteristics of the gels are summarized in Fig. 7a. One E5.5 gut was embedded in each gel and the final GDNF concentration was kept constant at 10 ng/mL. Migration distance was evaluated from microscopy images (Fig. 7b) by measuring the average distance of the ENCC wavefront from the ileum wall (Fig. 6a). Two independent experiments were carried out (Fig. 7c,d) independently for all seven gels, and an additional independent experiment for a subset of gels (Figure S8).

The breadth of the ENCC halo was strongly correlated with gel stiffness, decreasing monotonously with increasing gel stiffness (Fig. 7b-d). Migration distance was always greatest in the softest (150 Pa) gel; migration was either completely halted (Fig. 7d) or delayed by at least 20 hours and restricted to a small (up to 80 μm) region around the gut (Figure 7c) in the hardest gels, the 5 kPa (no. 6) and 15 kPa (no. 7) gels. For intermediate elasticity values (540 Pa–3000 Pa), migration distances were intermediate between these two extremes. Remarkably, in this range of elasticity, two pairs of gels that had very similar elastic moduli (nos. 2&3 and nos. 4&5) but differed strongly in porosity and collagen content (the less porous gels had a higher collagen concentration, see Fig. 7a) yielded similar migration distances (detailed comparisons for these gels are shown in Figure S9). Thus, ENCC migration is strongly modulated by and correlated with environmental stiffness.

At a given collagen concentration, migration distance was dependent on gel fiber structure (which, in turn, determines elastic modulus and porosity). Indeed, gels 1 and 6 had identical collagen concentrations of 2 mg/mL (Figs 7c,d & S10) but yielded migration distances of 600 μm (gel 1) or 0 (gel 6, higher fiber thickness). In the specific case in which collagen concentration is varied but pH and gelling temperature are kept constant, the Young modulus increases linearly with collagen concentration (as found here and in Wolf *et al.*^48^). From the results presented above, it follows that in this case ENCC migration distance from gut explants decreases with increasing collagen content (as can be seen by comparing gel pairs 1&2 and 3&4). Such a decrease has been reported for NCCs migrating from neural tubes in 3D collagen gels without GDNF^47^,^49^.

Migration distance did not depend on porosity alone, as gels with similarly low porosities (gels 1, 2, and 4) yielded different migration distances (Figs 7c,d & S9). The softer gels (gels 1&2) used in our study tended to have very small pores (mostly < 1 μm), whereas the stiffer gels (gels 6&7) had larger pores (mostly > 3 μm). Remarkably, these larger pores did not favor ENCC migration.

### Proteolysis is required for ENCC migration in collagen gels

**Figure 8:**
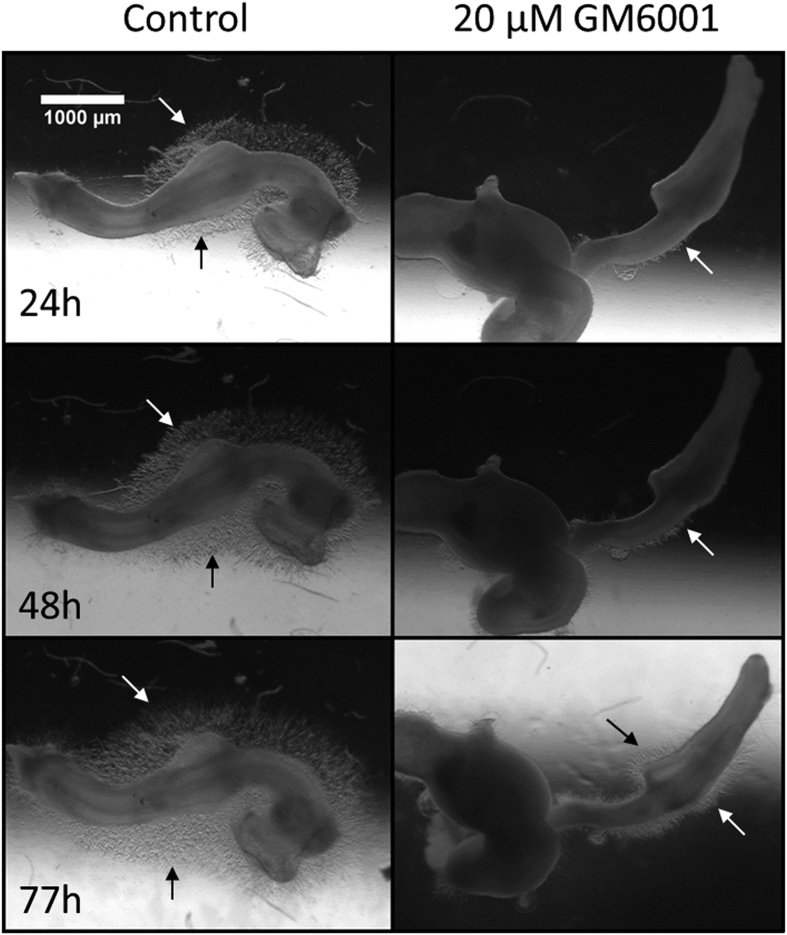
Representative (*n* = 4) effect of 20 μM GM6001 on ENCC migration. E5 gut embedded in a 150 Pa gel, with 10 ng/mL GDNF, with or without GM6001. Arrows indicate the position of the ENCC migratory front.

It has recently been shown that matrix metalloproteases (MMPs) are essential for enteric^50^, trunk and cervical^51^ NCC migration. We therefore investigated whether the observed dependence of ENCC migration distance on collagen gel stiffness was dependent on the collagenase-type enzymes produced by NCCs. We inhibited MMPs with GM6001 (also called ilomastat). A representative result is shown in Fig. 8.

For all guts tested (*n* = 4), GM6001 inhibited ENCC outgrowth, as shown by comparisons with untreated guts (n = 4). We confirmed that GM6001 did not affect other cell motility functions unrelated to proteolysis: it had no effect on ENCC migration on 2D substrates (Figure S11) at the same concentration (20 μM). Thus, the ENCCs actively degrade the collagen gel matrix in order to progress. It should be noted that 3D cell migration in collagen gels has been shown to occur with^48^ or without remodeling/degradation of the gel matrix^52^, depending on the cell type considered.

## Discussion

On 2D substrates, ENCCs migrated over a large range of stiffnesses, from several hundred Pa to 1 MPa. One outcome of our result is that ENCCs can interact, spread and migrate on very soft surfaces (100–200 Pa) unlike mesenchymal cells that originated from the same explants. This correlates with the ability of ENCCs to colonize embryonic gut tissue presenting a similar range of elasticity, as revealed by our measurements. It indicates that these cells may be particularly well adapted to migrate on soft environment. This probably reflects differences in intrinsic cell elastic properties, contractility or to adhesion receptor repertoire, and their ability to adapt the migratory machinery to the elasticity of the 2D environment. The difference of behavior between ENCCs and mesenchymal cells to substrate elasticity is most likely related to cell-type. Previous studies have for example shown that fibroblasts and endothelial cells exhibit a round-shape on very soft surfaces while neutrophils can spread^53^,^54^. Glioma cells exhibit a dramatic reduction in the speed of locomotion on softer substrates^24^, whereas neurons produce more branched neurites^55^.

In 3D gels, elasticity was found to be an accurate predictor (Fig. 7b,d) of ENCC migration distances, hinting at a possible underlying physical mechanism: to progress within the gel, the cells have to “push” collagen fibers aside, deforming or even remodeling the gel locally. The energetic demand on the NCCs should increase with the stiffness of the medium or fiber thickness. However, in addition to this process, our experiments with MMP inhibitors showed that proteolysis of the surrounding matrix was also required for ENCCs to migrate in collagen gels. Thus, for cells migrating into a dense collagen gel, the time it takes for the collagenase enzymes to degrade the matrix will clearly be proportional to collagen concentration, but will also depend on collagen fibril structure and the area accessible to the enzymes. MMPs are probably more efficient at degrading high surface-to-volume ratio collagen fibers, such as those present in weakly reticulated gels (gels 1&2), and this would probably affect the progress of cells in gels containing thick collagen fibers (gels 6&7). The slower migration of ENCCs in stiffer gels can thus be explained in terms of biochemical and/or physical effects, with both probably contributing to some extent. Anderson^50^ demonstrated that the ENCC migration speed decreases in mouse hindgut when MMPs are inhibited by GM6001, further stressing the importance of ECM cleavage for ENCC migration. We note that zinc-protein inhibition by GM6001 also affects the signaling pathways Notch^56^, ephrin^57^ and EGF signaling^58^; Ephrin signaling is involved in cell-cell repulsion following engagement, which is important for coordinated migration and neuronal axon guidance^59^.

We found that gel porosity alone was not correlated with migration distance. This result may seem at odds with recent findings by Wolf *et al.*^48^ suggesting that pore size and nuclear deformability are the factors determining cancer cell migration in collagen matrices. We highlight several differences that may explain this discrepancy: the cancer cells studied by Wolf *et al.* were less deformable than ENCCs as they could not penetrate filter pores smaller than 2–3 μm^48^, whereas NCCs have been shown to pass through pores of less than 0.9 μm in diameter^60^. As the sizes of the pores used here and in their work were similar (1–10 μm), it seems reasonable to suggest that pore size may be a limiting factor for the cells studied by Wolf *et al.* but not for ENCCs. Another important difference lies in the fact that Wolf *et al.* calculated cell speed from the total path length (including go, stop and reversal phases) extracted from single-cell to-and-fro trajectories, whereas we followed the ENCC migratory front position as a function of time. Finally, we cannot rule out the possibility of differences between cell types. Newgreen^60^ studied the influence of physical parameters of the ECM on NCC migration and suggested that the effective size of the microspaces between fibrils in the ECM were determinants of NCC migration. We show here that increased fibril organization (increased fibril length, thickness, and connectivity of the collagen fiber network), which is directly related to stiffness, determines migration speed, whereas pore size, at least within the 1–10 μm range, does not. In addition to providing fundamental insights into the mechanisms of ENCC migration, our results highlight the need to control the gel formation process precisely (in particular gelling temperature and pH) when comparing the outcomes of 3D migration assays.

Tensile testing showed that the *in-vivo* bulk elasticity of the mesenchyme was in the range 100–1000 Pa (for chick) and 1–3.5 kPa (for mice), corresponding to gels 1–4 and gels 4–6, respectively (Fig. 7a). During the development of both chicks and mice, tissue stiffness increases with gut age during the period of ENCC colonization, such that the elasticity perceived by the migrating ENCC in the hindgut is two to three times that perceived in the midgut. AFM indentation provides an estimate (the probe is larger than ENCC protrusions) of the force that the ENCCs must apply to penetrate the mesenchyme. We found that this force increased by a factor ~6 with increasing gut age from E4.5–E5 to E8 (Fig. 2b), consistent with the results obtained for tensile testing. SHG imaging revealed that embryonic gut stiffening was linked to a gradual process of collagen enrichment and organization. SHG signal intensity was found to be uniform in the gut up to E6 (E12.5 for mice), corresponding either to a non-specific background signal (noise) or to short, uniformly distributed collagen fibrils. At E7–E8 (E14.5 in mice), distinct, circularly-arranged collagen fibers appeared throughout the gut in both chicks and mice (Figs 3 and 4b, S12–13). Collagen fiber density was found to increase in the chick hindgut from E6 to E8 (Fig. 3). As we demonstrated *in vitro* that increasing stiffness, within the physiological range (100 Pa–5 kPa) by increasing collagen fibril organization slows down ENCC progression in 3D (Fig. 7), and that ENCCs must actively degrade collagen to progress (Fig. 8), our results suggest that distal tissues may be harder to invade because they are reached at a later stage and are thus stiffer. Using mice guts with fluorescently labeled ENCCs, Druckenbrod *et al.*^61^ measured the speed of ENCCs in the different regions of the gut. They found that the average speed of the ENCC wavefront in the ileum and in the hindgut are respectively 45 ± 10.8 μm/h (n = 4) and 30 ± 7.8 μm/h (n = 5), i.e., the migration speed in the hindgut is 33% lower than that in the ileum. We found that the stiffness of the ileum at E12.5 is 1452 ± 253 Pa (n = 8) while the stiffness of the hindgut at E13.5 is 2551 ± 524 Pa (n = 5), i.e., the stiffness at the level of the ENCC migration front increases by 75% between E12.5 and E13.5. The inverse correlation between stiffness and migration speed we found in collagen gels (Fig. 7) is therefore also qualitatively observed *in-vivo*, suggesting that the decrease of the ENCC migration speed in the distal parts of the gut may be due to developmental stiffening. We cannot exclude that other factors, such as a different mechanism of chemoattraction, or an endothelin-3 mediated effect may play equally important roles in explaining why the ENCC migration speed decreases in the hindgut. We note that our observations are also potentially relevant for ENCCs invading the colon via the transmesenteric pathway^62^, as increased stiffness of the midgut and hindgut may prevent the cells from crossing the midgut-mesentery and mesentery-hindgut junctions.

ENCCs depleted of ß1-integrins cannot colonize the hindgut, resulting in a Hirschsprung-like phenotype^11^. Integrins are ECM mechanoreceptors that have been linked to MMP- and adhesion-based migration in 3D substrates. It is therefore likely that ENCCs must exert a traction force on the ECM via integrins to progress in the hindgut, a phenomenon also observed when ENCCs invade collagen gel (Figure S5). Our results suggest that the need for this traction force may be particularly important at late stages of ENCC invasion, because of the comparatively high stiffness and collagen density of the hindgut.

In addition to conditional mouse mutants in which NCCs are depleted of ß1-integrins, several mouse models of Hirschsprung disease (edn3/EDNRB^63^, Sox10^13^) display a delay, by about one day, in the chronology of ENCC migration with respect to the wild type. Thus, they reach the proximal hindgut at E13.5, rather than E12.5. In one study^63^ in which young ENCC donor guts were grafted onto older ENCC-free host hindguts, the one-day-older gut was completely impervious to ENCC, suggesting the existence of a “time window” for ENCC colonization, that could be due to developmental stiffening of the hindgut (Fig. 4a). However, in another study^64^, ENCCs were able to colonize older hindguts, although the speed of migration decreased in proportion with the increase in host gut age. Additionally, a haploinsufficient mouse model (Tcof1^+/−^) 65,66 has recently been shown to present an ENCC migration delay of about one day whilst displaying complete ENS formation at late stages of development. It therefore seems that ENS formation can be completed even in old hindgut, unless the Ret^64^ and Tcof1^+/−^ 65 mutations otherwise affect ENCC migratory pathways, mechanosensing or the properties of gut tissues. It seems reasonable to conclude that developmental stiffening may contribute to the slowing of ENCC progression and aggravate conditions in which the ENCC progenitor pool is already affected in other ways^64^,^65^ (reduced proliferation, adhesion and/or early differentiation).

Our findings provide new insight into ENS ontogenesis and additional mechanisms likely to contribute to the NCC migratory defects underlying neurocristopathies^67^, such as Hirschsprung disease. In about 50% of Hirschsprung disease cases, the underlying cause is unknown^68^. The observation that proteolysis is required for ENCCs to invade collagen gels (Fig. 8) and chick gut *in vivo*^50^ indicates that identifiable candidate genes could include genes encoding MMPs or regulating the mechanotransduction response of NCCs. Futures studies aiming to determine whether tissue stiffness is a limiting factor for the speed of ENCC migration *in vivo*, could investigate the direct correlation between local tissue stiffness and ENCC speed in the gut, in mice with fluorescently labeled ENCCs. Methods to soften the tissue could help to improve the outcome of cellular therapies^69^,^70^ based on NCC transplantation in the aganglionic gut, in neonates with a high stiffness of mature tissue likely to slow or halt cell migration. Understanding the physical limits of NCC will finally further our understanding of cancer^71^, as the invasive cell behavior essential for normal embryonic development can lead to deleterious tissue invasion and metastasis in adults.

## Materials & Methods

### Animal models

Fertilized chicken eggs were purchased from Centre Avicole d’Ile de France (CAIF, France) and incubated at 37.5 °C for up to 9 days. Fertilized quail eggs were purchased from “Les cailles de Chanteloup” (Corps-Nuds, France) and incubated at 38 °C for 5.5 days. Exact staging of the guts was achieved by morphological comparisons, as previously described^72^. Precise staging was required, because the position of the ENCC migratory front changes over both space and time. We used the results of a previous study^29^ to infer the approximate position of the ENCC migratory front.

The lethal spotted (Edn3^ls/+^) mouse has been described elsewhere^73^. Lethal spotted mice were crossed to produce Edn3^ls/+^, Edn3^ls/ls^ and wild-type embryos, and age was determined by considering the detection of a vaginal plug to correspond to E0.5. Experiments were performed in accordance with the ethics guidelines of the INSERM and CNRS and were approved by the Committee on the Ethics of Animal Experiments of the Institut Curie (National registration number: #118).

### Sample preparation

The digestive tract was dissected and the mesentery was completely removed. The dissected guts were either used as such (tensile testing), or embedded in collagen gels (migration assays) or low-melting point agarose gels (Type VII, Sigma-Aldrich) at 37 °C. For AFM and SHG analyses, the guts were embedded in agarose and a double-blade cutter (Multirex, France) was used to cut 1 mm-thick transverse slices from different parts of the gut. Slices were immobilized by “gluing” them onto the base of a Petri dish with liquid agarose. The samples were hydrated with PBS, kept at 4 °C, and imaged within 5 h by AFM or SHG microscopy.

### Uniaxial tensile test

The method we use is described in a report by our group^26^. The gut was placed in a rectangular, optically transparent tank filled with PBS and immobilized at its anal end by pinning. The stomach was inserted into a hook formed at the end of a glass fiber (length ~10 cm, diameter ~ 15–20 μm) and the gut was sprinkled with carbon particles for particle image velocimetry (PIV) analysis. The glass fiber was produced by pulling a heated Pasteur pipette to a length of about 10 cm and the hook was formed by applying heat to end of the pulled pipette. Before measurement, the bending stiffness of the fiber was measured in air, by placing it in a horizontal position and hanging small weights (pieces of known lengths of a thin plastic wire of known linear mass) at its end. The force-angular deflection characteristic was linear over the 0–30° range and yielded a sensitivity 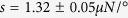 (per degree of deflection of the end of the fiber). The same fiber was used for all measurements reported in this study, and the forces applied were in the range 0–20 μN (angle: 0–15°). Stress was calculated by dividing the force applied by the local section area of the gut. The mean radius of the gut in different regions was measured on high-magnification images, to infer the local cross-section *S* = *πr*^2^. We did not consider the cross-sectional area of the lumen, because it accounts for less than 5% of the total gut cross section at early stages of gut development (see Fig. 3). The fiber enters the PBS-air interface at a 90° angle. We used high PBS levels (~15–20 cm) to eliminate the contribution of meniscus forces to fiber deflection. The fiber was secured at its other end to a μm-stepper motor. The speed of the motor (fiber displacement) was adjusted to the length of gut studied, to achieve a strain rate of ~10%/min for all guts. The deflection of the fiber and the deformation of the gut were monitored simultaneously, from the side, by a camera. The deformation of each region (HG, ileum, jejunum) was calculated by PIV (Tracker plugin for ImageJ, courtesy of O. Cardoso) for the carbon tracers.

For collagenase tests, guts were treated with 1 mg/mL (50–200 U/mL) collagenase type IV (Life Technologies) in PBS supplemented with 0.9 mM CaCl_2_ and 0.5 mM MgCl_2_

### Atomic force microscopy (AFM)

AFM elasticity maps were obtained with a Nanowizard I (JPK Instruments, Germany) instrument equipped with a 100 μm *z*-piezo module (CellHesion) and a liquid-cell-adapted cantilever holder. The cantilever probes (Novascan, USA) were functionalized with a spherical (10 μm diameter) borosilicate glass indenter and had a typical stiffness of ~60 mN/m, as measured by the thermal tuning procedure in air. The stiffness of the cantilevers was further confirmed by the reference spring cantilever calibration method and was found to be consistent, to within 10%, with the results of the thermal tuning procedure. Force curves were collected in PBS at room temperature, at a constant indentation speed of 50 μm/s and a maximum indentation force of 1 nN, such that the indentation depth was less than 5 μm (the radius of the indenter). The elastic modulus was obtained by performing Hertz fits of the indentation force against depth curves. Elasticity maps were obtained by scanning between 20 × 20 (400 force curves) and 40 × 40 points (1600 force curves) on a 100 × 100 μm area. The resolution of the scans was therefore between 2.5 and 5 μm. The position of the tip relative to the sample was recorded with a CCD camera at the beginning and end of the scan, to ensure that no substantial drift of the sample had occurred during the duration of the scan. The low-quality pictures of the CDD camera were then compared with the high-quality images of the gut sections obtained by microscopy (Nikon, 10X or 20X), and the elasticity maps were superimposed onto the microscopy images. We typically obtained 5–10 elasticity maps for any given sample. The mean elasticity of the mesenchyme was calculated by averaging the elastic modulus obtained over the area occupied by the mesenchyme. Extreme values of the modulus resulting from measurement or fitting problems were excluded from the calculation of the average.

### Second-harmonic generation (SHG) imaging

SHG images of collagen in chick gut sections were obtained with an upright Leica SP5 microscope (Leica Microsystems Gmbh, Wetzlar, Germany) coupled to a femtosecond Ti:sapphire laser (Chameleon, Coherent, Saclay, France) tuned to a wavelength of 810 nm for all experiments. The beam was circularly polarized. We used a Leica Microsystems HCX IRAPO 25x/0.95 W objective. The SHG signal was detected in epi-collection through a 405/15-nm bandpass filter, by an NDD PMT (Leica Microsystems), with a constant voltage supply, at constant laser excitation power, allowing the direct comparison of SHG intensity values.

### 2D organotypic cultures

Polyacrylamide hydrogels (PAA) were produced 24 h before culture, according to a previously established protocol^74^. PAA gel surfaces, used for 2D cultures, were treated with sulfo-SANPAH (0,2mg/mL, Pierce Biotechnology), activated with 365-nm UV light source, rinse with HEPES several times and then incubated with bovine plasma fibronectin (FN, Sigma) at a final concentration of 20 μg/ml in PBS and incubated overnight at 37 °C.

Uncoated PAA hydrogels were stored in PBS at 4 °C for at least 24 h, to equilibrate, before atomic force microscopy (AFM). PAA Young’s moduli were determined at room temperature in PBS. Up to four independent samples were studied. Elasticity maps, for five different regions per sample, were obtained by scanning 4 × 4 points (16 force curves) or 8 × 8 points (64 force curves) of a 100 × 100 μm area. Mean elastic moduli ( ± SEM) of 1976.3 ± 287.7 Pa, 220.5 ± 57.7 Pa and 120.6 ± 41.0 Pa were obtained for gels containing final acrylamide/bis-acrylamide percentages of 10%/0.03%, 3%/0.06% and 3%/0.03%, respectively.

Two-dimensional organotypic cultures of E5.5 quail midgut explants were performed on the glass bottoms of Ø = 35 mm μ-Dishes and Ø = 35 mm μ-Dish elastically supported surfaces (ESSs) of 28 kPa, 15 kPa and 1.5 kPa (Biovalley) coated overnight with FN (20 μg/ml, Sigma). E6.5 chick midgut explants were cultured on FN-coated PAA hydrogels. Culture was performed overnight in DMEM/F12 (Gibco) medium supplemented with insulin-transferrin-selenium (Gibco), in a damp atmosphere containing 5% CO_2_/95% air, at 37 °C.

### Immunostaining and microscopy

Cultures were fixed by incubation with 4% paraformaldehyde in PBS for 10 minutes at room temperature. Cells were permeabilized by incubation with 0.5% Triton X-100 for 5 minutes at room temperature before blocking with 30 mg/mL bovine serum albumin (BSA, Sigma) in PBS. Cultures were first immunostained with an antibody against NC1 (mouse IgM, produced in-house, diluted to 10 μg/mL) and a Cy3-labeled donkey anti-mouse IgM secondary antibody (Jackson, diluted 1/300). Cultures were then blocked by incubation with control anti-mouse IgG for 20 minutes in the dark at room temperature and immunolabeled with Alexa Fluor 488-labeled mouse anti-TUJ1 (class III beta-tubulin) antibody (Covence, diluted 1/500) and with 4′,6-diamino-2-phenylindole (DAPI, Molecular Probes, diluted 1/1000). Fluorescence image acquisition was performed on an epifluorescence-histology microscope (Eclipse 90i Upright) at the Nikon Imaging Centre @ Institut Curie-CNRS. This microscope was equipped with a CCD Camera (CoolSNAP HQ2, Photometrics) and piloted with MetaMorph software. Entire explant images were reconstituted with Photoshop software, with the adjustment of brightness and contrast over the entire image to enhance fluorescence signals.

### Collagen gel migration assays

Our protocol was inspired by the work of Nagy *et al.*^7^. Collagen type I was obtained from rat tail tendon after acidic extraction, as previously described^75^. Collagen gels were prepared on ice by sequentially adding 20 mM acetic acid to collagen type I to dilute it to an appropriate concentration, together with M199 medium and 1 M carbonate-bicarbonate pH 9 to adjust final pH. Gelling occurred within 5 minutes of the neutralization of the collagen solution at room temperature of on a heating plate at 37 °C. Seven different collagen hydrogels were synthesized by varying the collagen concentration (2 or 4.5 g/L), gelling temperature (37 °C or room temperature) and final pH (6.5, 7 or 8.5). The stiffness of the collagen gels was assessed by shear rheology after 1 h of polymerization at the appropriate temperature with a strain of 0.1% at 1 Hz (MCR 302, cone/plate geometry of 25 mm, Anton Paar). We converted shear strength to Young moduli, assuming a Poisson ratio ν = 0.5. Pore size was determined from the second-harmonic generation stack (SP5 with objective x25, Leica, and pulsed Ti laser from Spectra-Physics), with Fiji software. Gel porosity and modulus (SEM < 10%) were highly reproducible.

The liquid collagen solution (1 mL) was poured into a custom-made, gut-sized cylindrical cell (Ø = 2 cm, height = 1 cm). Each chick gut was transferred, with the help of tweezers, to a separate cell. We straightened the gut as far as possible and made sure it was positioned in the bulk of the collagen solution before it gelled. After 45 minutes, a stationary state of polymerization was reached. We added 1 mL DMEM (4.5 g/L glucose with GlutaMAX, Life Technologies) supplemented with 1% by weight penicillin and streptomycin (Life Technologies) and glial-derived nerve growth factor (GDNF, R&D Systems, USA) applied to the top of the gel. The reagents quickly diffused into the bulk of the gel. The resulting embedded guts were then incubated at 37.5 °C and 100% relative humidity. The supernatant was replaced every 24 h with freshly prepared DMEM+PS+GDNF. Ilomastat (GM6001, Merck-Millipore) was added to the medium at a final concentration of 20 μM for gels and supernatants, as previously described^48^. Time-lapse imaging (1 image/min) was performed on an inverted microscope (Leica) or a binocular (Leica) microscope, at 37.5 °C. Cell migration images were obtained by removing the samples from the incubator at fixed time points (from 18 h to 115 h) and imaging them with a binocular microscope under transmitted light. The mirror was adjusted so the samples were illuminated in twilight (pseudo-Nomarski mode) to enhance the contrast of the migrating cells.

## Additional Information

**How to cite this article**: Chevalier, N.R. *et al.* How Tissue Mechanical Properties Affect Enteric Neural Crest Cell Migration. *Sci. Rep.* **6**, 20927; doi: 10.1038/srep20927 (2016).

## Supplementary Material

Supplementary Video 1

Supplementary Video 2

Supplementary Information
