# Supplementary material for: How Tissue Mechanical Properties Affect Enteric Neural Crest Cell Migration
Source: Sci Rep. 2016 Feb 18;6:20927. doi: 10.1038/srep20927 (PMC4757826; doi:10.1038/srep20927)
Supplement: Supplementary Information [file srep20927-s3.doc]

# Supporting information to “How Tissue Mechanical Properties Affect Enteric Neural Crest Cell Migration”

N.R. Chevalier, , E. Gazguez, L. Bidault, T. Guilbert, C. Vias, E. Vian, Y. Watanabe, L. Muller, S. Germain, N.Bondurand, S. Dufour, V. Fleury


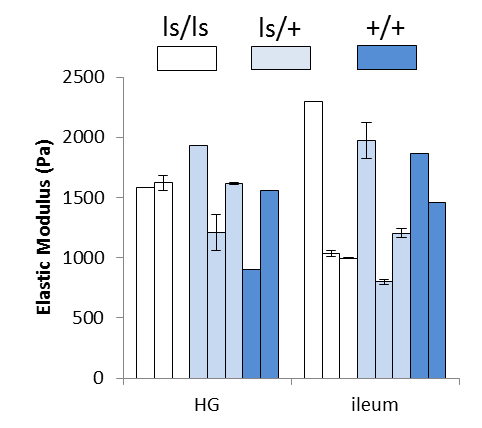


Figure S1: Elastic modulus obtained from tensile testing of the guts of ls/ls, ls/+ and +/+ from two different litters of E12.5 mice, at the start of hindgut invasion by ENCCs. For each gut, modulus in the hindgut (HG) and ileum were extracted; in one case (missing bar), the segment deformation could not be reliably computed. Error bars are SEM of 2-3 successive tensile tests, and are not indicated when only one test was performed. Average stiffnesses ±SD for the ls/ls, ls/+ and +/+ genotypes are: 1603 ± 26 Pa (n=2), 1587 ± 361 Pa (n=3), 1357 ± 465 Pa (n=2) for the hindgut and 1665 ± 890 Pa (n=3), 1325 ± 595 Pa (n=3), 1508 ± 285 Pa (n=2).


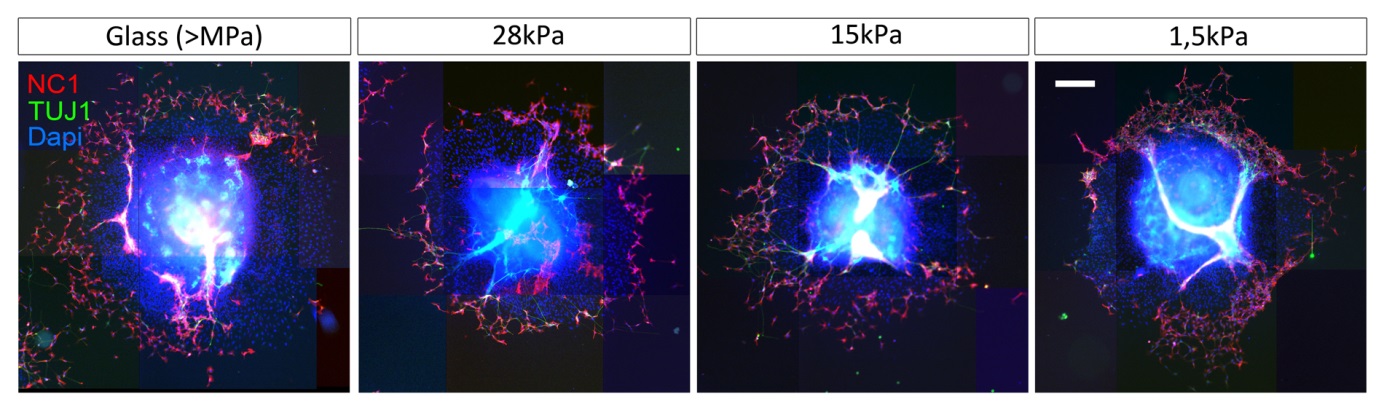


Figure S2: 2D migration assay of ENCCs on FN-coated substrates. Quail (stage E5,5) explants were cultured overnight on FN-coated glass and FN-coated PDMS substrates of different rigidities. The explants were immunostained for NCC marker (NC1, red), neuronal marker of ENCC (TUJ1, green), and Dapi (nucleus, blue). The number of explants analyzed for glass substrate, 28kPa and 15kPa is n=8 for each conditions and for 1,5kPa, n=7. Bar=200μm.


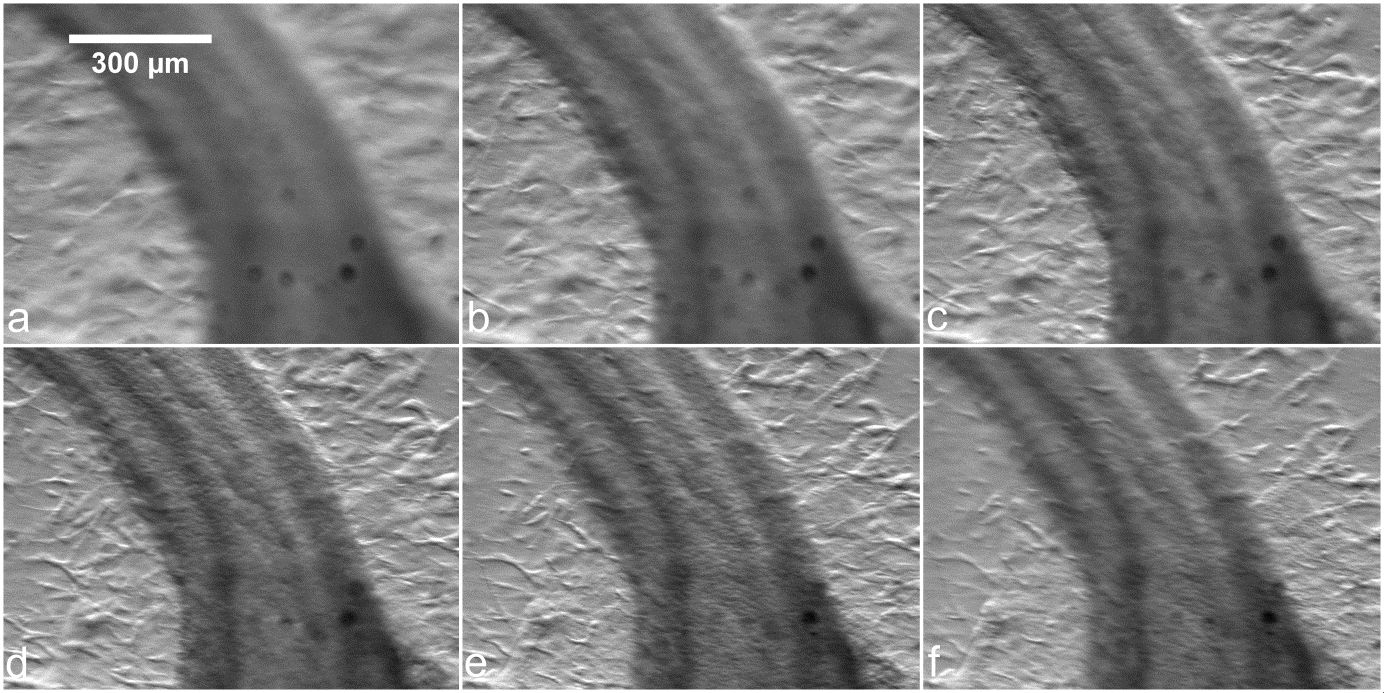


Figure S3 : High magnification view at different focal positions (a to f : below to above the gut) of the midgut of a E5.5 gut embedded in a 660 Pa gel, supplied with DMEM and 10 ng/mL GDNF, after 48H culture. ENCCs are distinctly seen below (a-b), in the plane of (c,d) and above the gut (e-f).

**Movie 1**

Accelerated (x3060) time-lapse of 17 h of a 1 image/minute time-lapse movie of the ileum of a HH28 chick gut incubated at 37.5°C on an inverted microscope. Time is counted from the moment the gut was imaged, i.e., approximately ~30min after embedding it in a 660 Pa gel supplied with DMEM and 10 ng/mL GDNF. The dark tubular segment is the post-umbilical midgut (ileum); the umbilicus is seen in the lower left corner while the upper right corner features a caecal appendix.

**Movie 2**

Accelerated (x8743) time-lapse performed for 12 h on a HH28 gut, 7 h after it was embedded in the softest collagen gel (E=150 Pa), at 10 ng/mL GDNF concentration and incubated at 37.5°C. Field size is 4.34 x 2.91 mm. The gut is imaged with a standard binocular in transmitted light but the mirror was set so it was illuminated in twilight: small thickness / refraction index differences such as those induced by the migrating ENCCs are thus revealed. Only the proximal hindgut is in focus.

The movie shows several interesting features of ENCC migration:

1. The more distal the ENCCs area located, the earlier they leave the gut, giving rise to the characteristic pear-shaped halo described in the main text.
2. No ENCCs migrate through the hindgut wall, however they are seen to exit through the cloaca (where the organ was sectioned from the embryo body)
3. Several bubbles were accidentally included in the gel upon gelation and get filled with liquid DMEM. These take the forms of circles, 5 of which are in contact with the gut (stomach-duodenum junction, duodenum, umbilicus, 2x HG). It is distinctly seen that the ENCCs travel faster in these collagen free regions and that they also pull on these defects and resorb them (especially visible on the defect close to the stomach), i.e., they remodel the gel matrix.
4. The fact that the ENCCs pull on the gel to migrate can also be evidenced by observing that impurities in the gel surrounding the gut are pulled towards the gut as the ENCCs progress. PIV tracking of the displacements of these impurities over a 1000 min period is shown on Figure S5.
5. Single “pioneer” cells or protrusions, that seems to explore the surroundings ahead of the ENCC migratory front are distinctly seen when zooming in the preumbilical gut region.


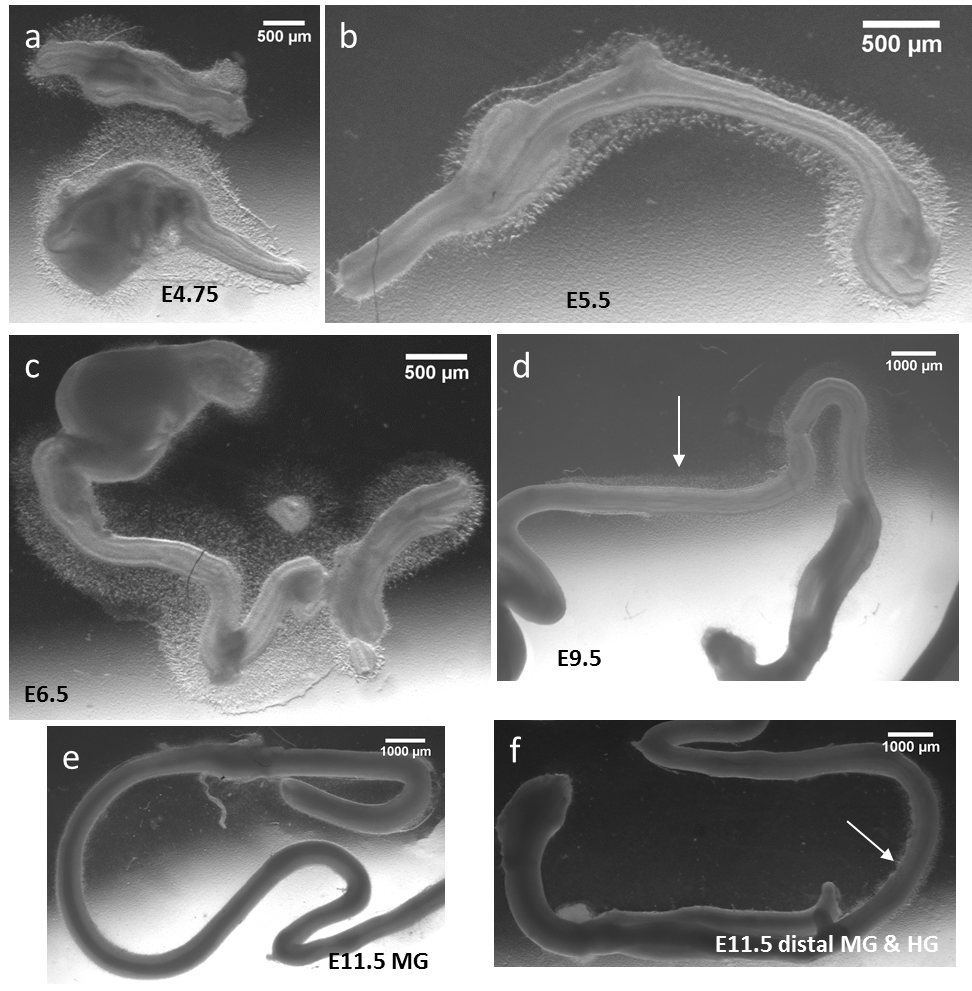


Figure S4: Effect of gut age on ENCC migration after 48h culture. All guts were placed in a 660 Pa gel and supplied with DMEM, final concentration of GDNF in gel & supernatant: 10 ng/mL. ENCCs migrate extensively throughout for 4.75 (a), 5.5 (b) and 6.5 (c) day old guts. At 9.5 days (d), migration still occurs but with a 1 day of delay. The ENCCs are seen to form a cone (white arrow) that extends in the rostro-caudal direction, confirming that the more rostral cells depart later, probably because of a delayed response of these more mature cells to GDNF signaling. For 11.5 day old guts (e-f), migration is strongly delayed and only occurs in a very restricted region of the ileum (white arrow). In all cases, even when the hindgut has been colonized by crest cells (d-f), cells never emerge from the wall of the hindgut. MG: midgut; HG: Hindgut.


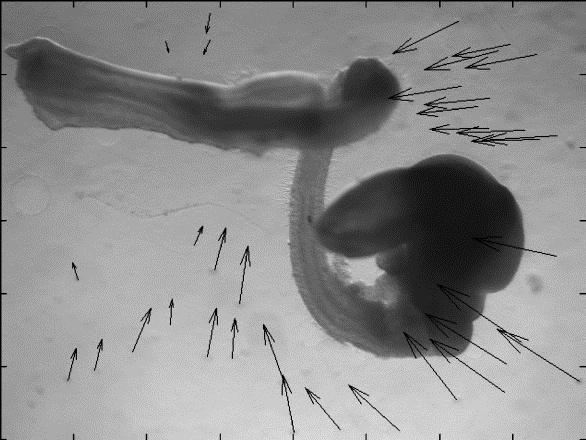


Figure S5 : Displacements of impurities in the gel during migration of ENCCs out of the gut, computed by PIV. The gel is pulled towards the gut as ENCCs migrate out the gut, into the gel.


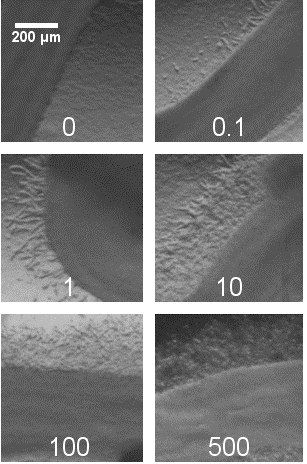


Figure S6 : Effect of GDNF concentration on ENCC migration, in units of ng/mL, for same age E5.5 guts in 660 Pa gels, after 48 h, close-ups around the midgut are shown. Migration distance and the density of the ENCC migratory front increase from 0 to 10 ng/mL, and then saturate above 10 ng/mL.


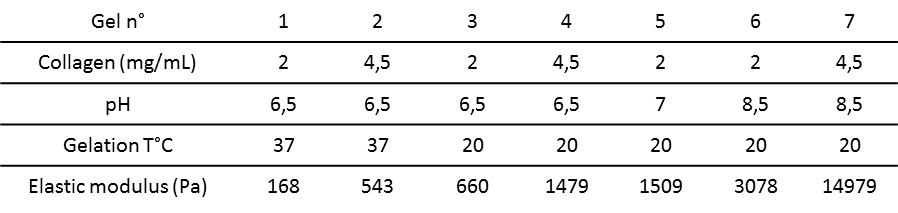


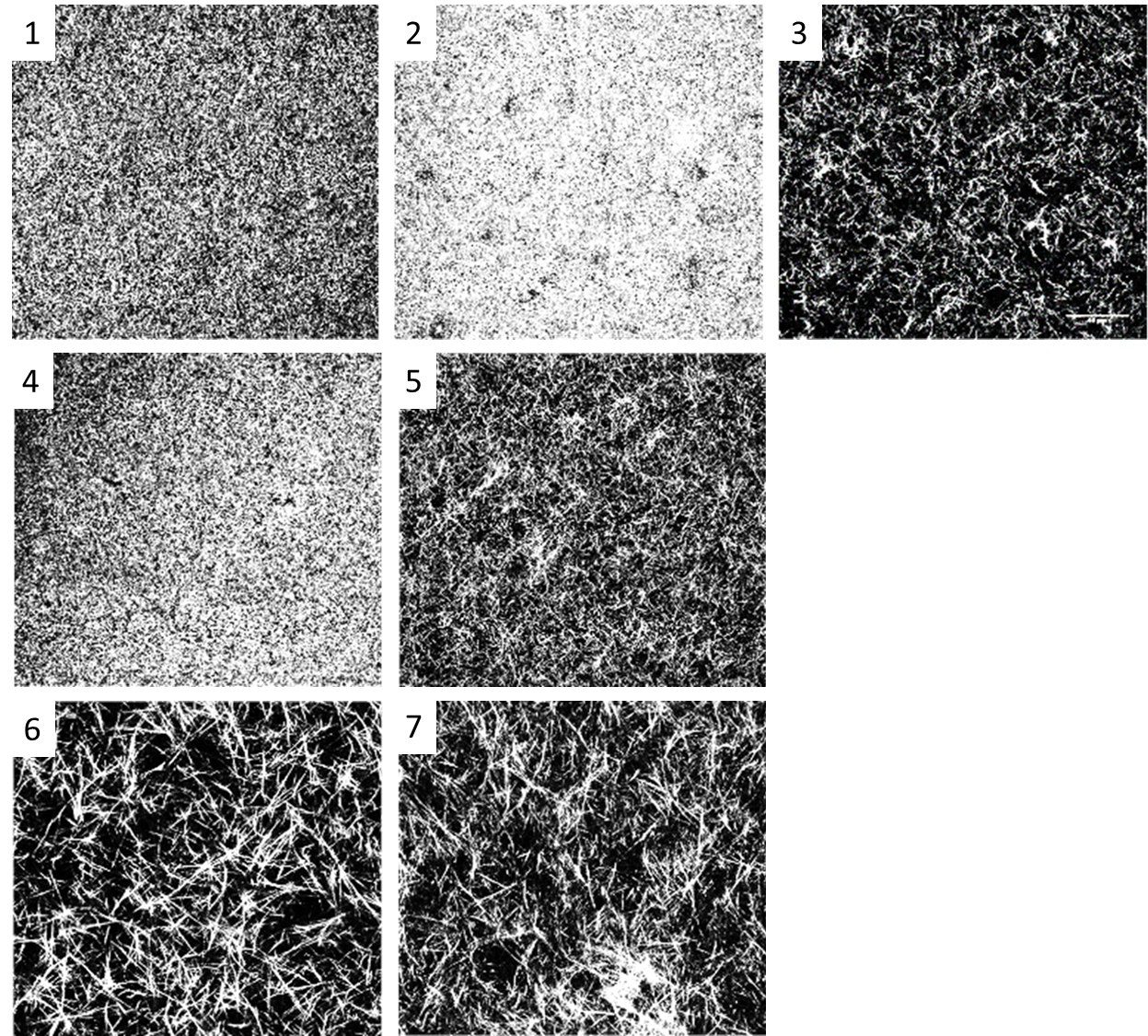


Figure S7: Collagen gels: collagen content, pH and temperature of gelation and resulting elastic moduli of gels and collagen fiber organization as imaged by SHG. The field of view of each image is 295.2 x 295.2 µm.


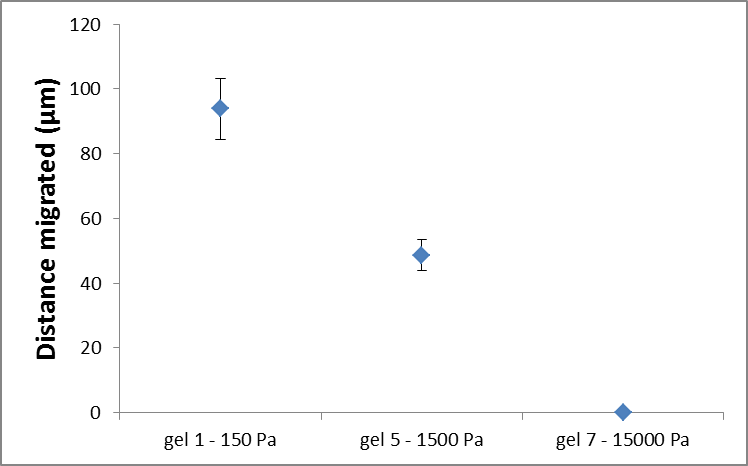


Figure S8: Independent migration assay tested for a subset of gels (gel n°1, 5 and 7) after 20h culture, confirming the trend on the influence of stiffness of Figure 7, main text.


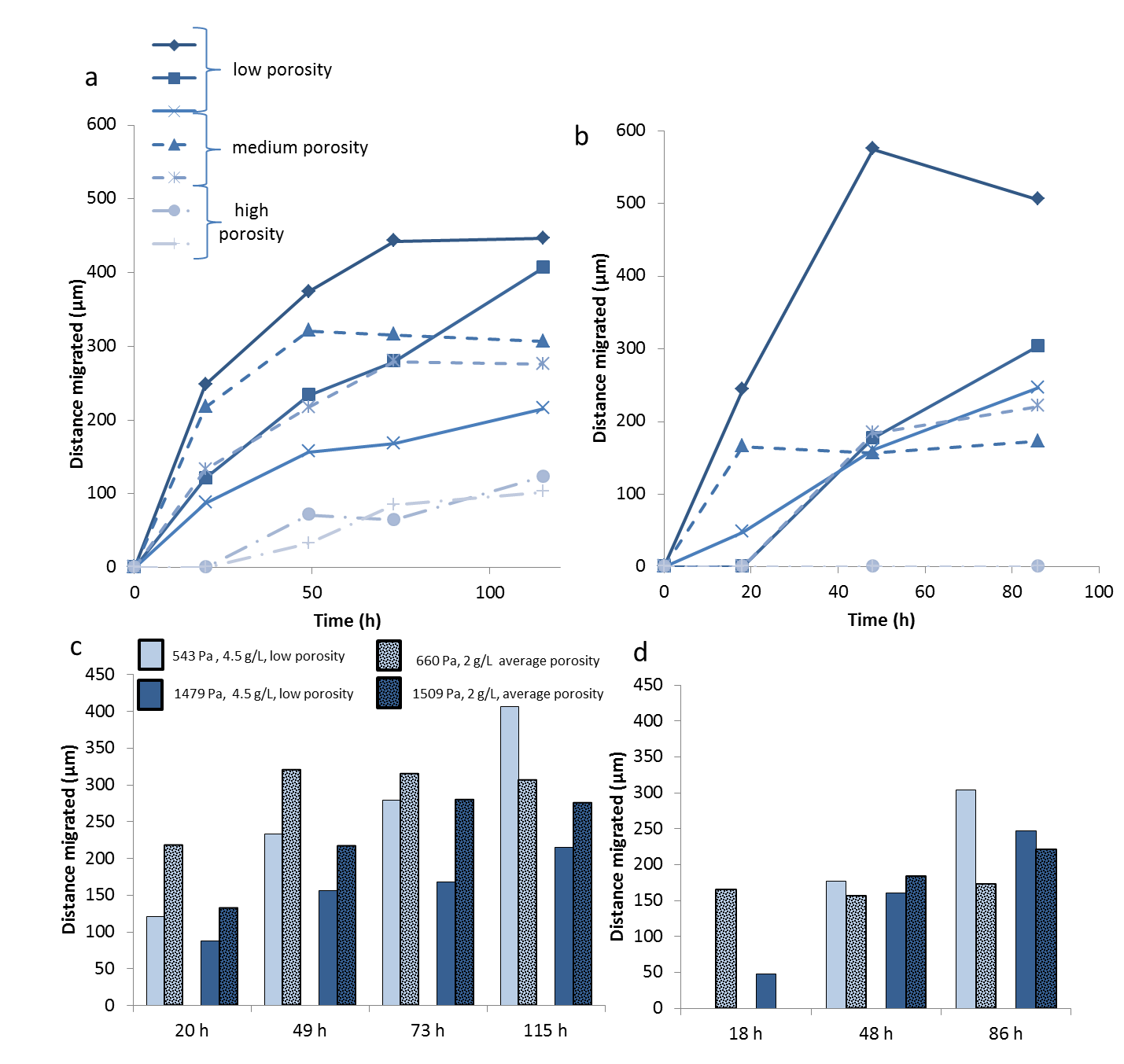


Figure S9: Effect of porosity for all gels (a-b) and plotted as bar histograms only for the two pairs of gels with equal stiffness but differing porosity (c-d). a-b) The gels can be classified in three categories: low porosity (gels 1,2,4 - solid lines), medium porosity (gels 3 & 5, dashed lines), high porosity (gels 6&7, dash-dot lines). Migration distances are plotted versus time for the two experiments performed (a, b). A direct correlation with porosity cannot be established. High porosity alone does not promote ENCC migration (gels 6,7), nor does very low porosity prohibit ENCC migration (gels 1, 2,4). .


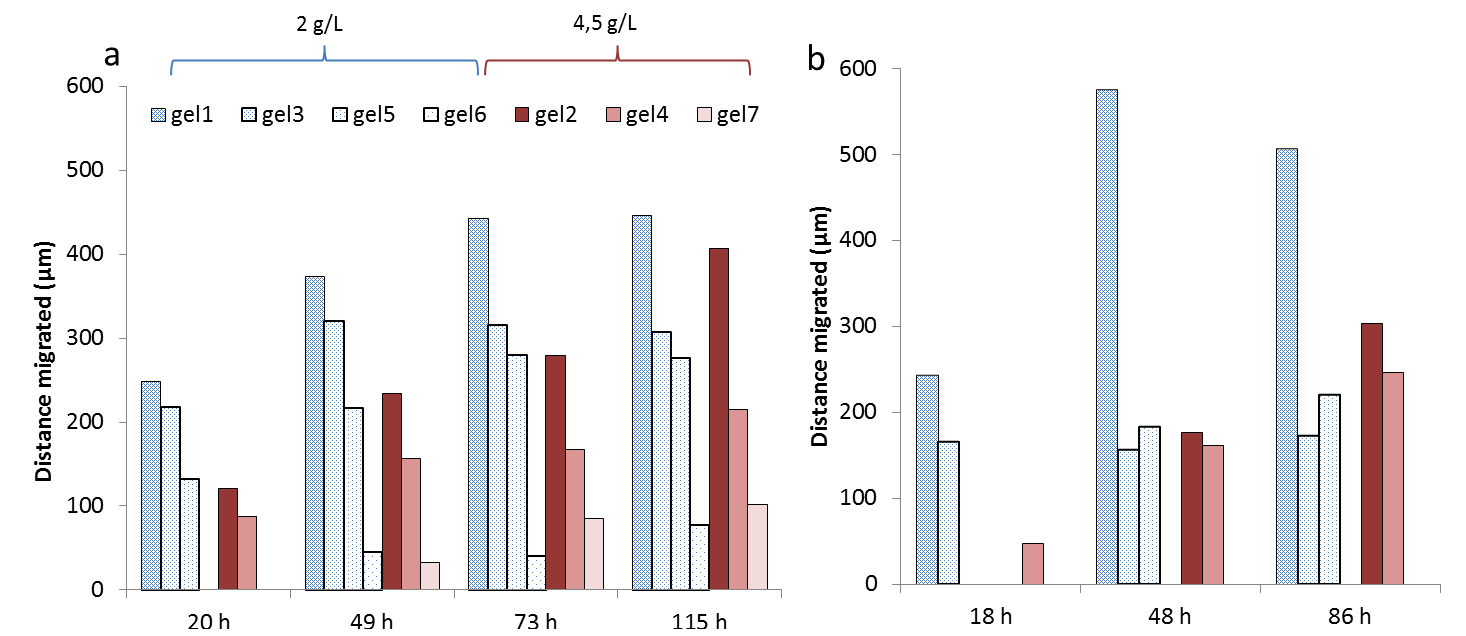


Figure S10: Effect of collagen concentration on migration distance. Two different collagen concentrations were used 2g/L (hashed bars, 4 different gels) and 4.5 g/L (full bars, 3 different gels). For each concentration, the gels are sorted from left to right ion order of increasing elastic modulus. For a given concentration, the decreasing migration distance vs. elastic modulus trend is clearly seen.


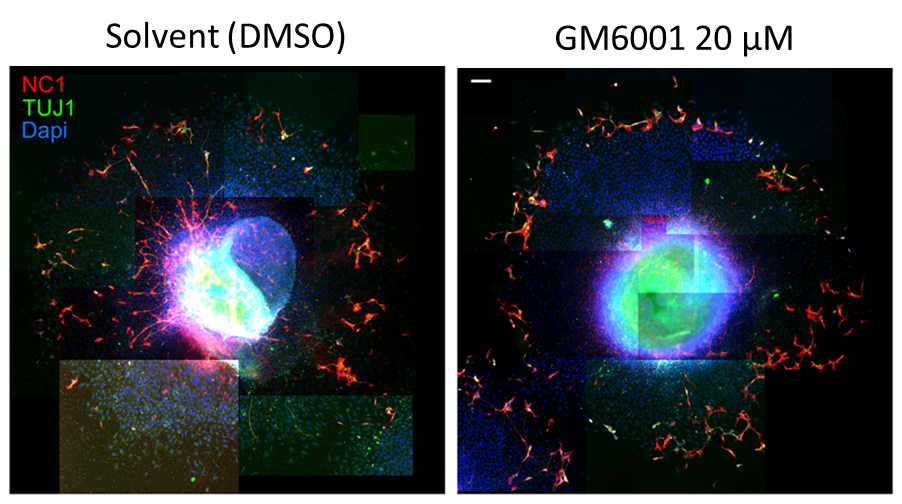


Figure S11: 2D migration assays in the presence of GM6001. E5.5 chick midgut explants were cultured over night with GM6001 (20 µM) dissolved in DMSO or as a control only with DMSO. Reconstituted pictures of the explants immunostained with NC1 (red), TUJ1 (green) and Dapi (blue) show that GM6001 does not seem to alter ENCC migration in 2D. A similar ENCC outgrowth was present in all conditions (n=4 explants analyzed for each conditions). Bar= 100µm


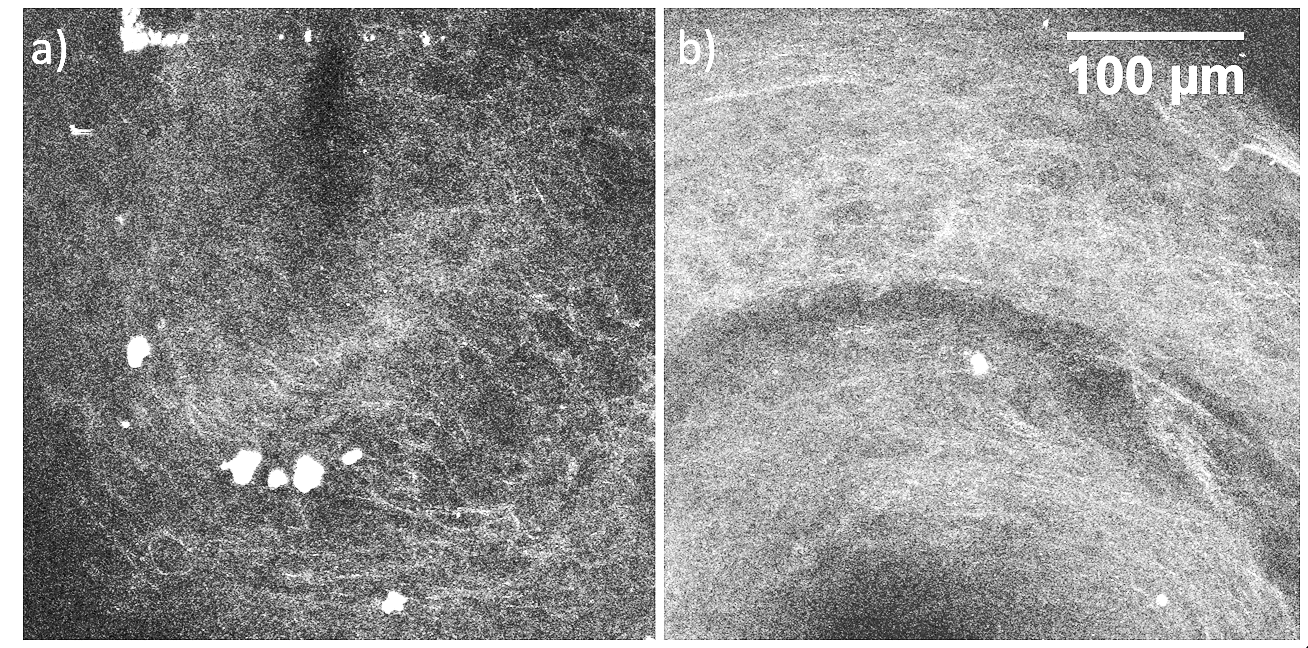


FigureS12 : SHG images on a different batch of eggs of chick hindgut at age a) E6 and b) E8. Acquisition parameters are equal between these two images. The SHG signal intensity increases with age in the hindgut; the fibers also become more organized in concentric rings.


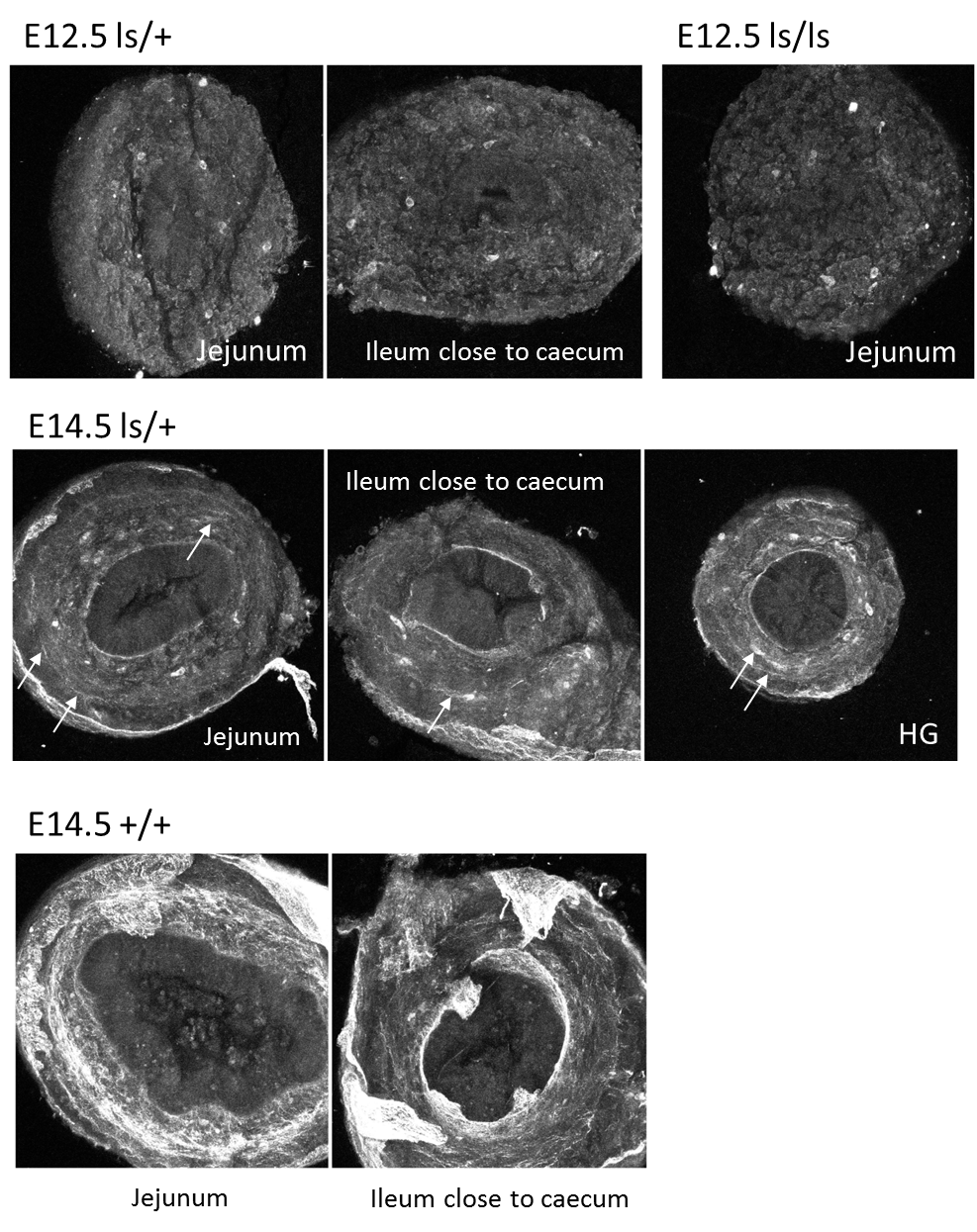


Figure S13: Full data set of mouse gut SHG images at E12.5 and E14.5, the genotype and section position along the rostro-caudal axis is indicated. The images are maximum z-projections of 3 µm-step z-stacks over a total depth of 100 µm from the section surface. All the acquisition parameters of the SHG microscope were kept constant between samples so that SHG signal intensities could be compared across ages and gut regions. White arrows on E14.5 ls/+ point out circularly arranged collagen fibers which are very distinctly seen on the E14.5 +/+ sample, but are not present at E12.5.
